# Supplementary material for: Integrating traditional medicine into antimicrobial resistance education: Community-centric preparedness in Zimbabwe
Source: PLOS Glob Public Health. 2025 Oct 27;5(10):e0005245. doi: 10.1371/journal.pgph.0005245 (PMC12558487; doi:10.1371/journal.pgph.0005245)
Supplement: S1 Data — (PDF) [file pgph.0005245.s001.pdf]

Parirenyatwa workshop

[Speaker 2]
A conversation on this notion of integration of traditional medical practice and conventional intervention in Zimbabwe, and especially around where does the approach to traditional medical practice sit, how is the approach to traditional medical practice, and also where does the healthcare system sit, what kind of system is it that we are supposed to operate in this relationship between the traditional medical practice and the conventional medical practice. So we maybe can start with that.

We can start with that anyway. And maybe we can start with the first question. How is traditional medical practice approached?

If these are your most generalised categories of research, it should just be supported by that. So maybe we can start with that question.

[Speaker 3]
So the first question I think, officially, it is somewhere on the paternalistic spectrum, considering that our government is now trying to include the traditional medicine, herbal use, and try to find more about it, how it helps us. So I think they are trying so much to be partners with the traditional healers, even though some of them are still a bit sceptic and conservative and a bit shy about it, because they can't themselves explain how it helps us.

[Speaker 5]
I would say it is marginalised, because inasmuch as we have knowledge on traditional medicine and how they practice it, our health sector has still begun the convention of the western medicine. So even if you go to the hospital, we are still going to prescribe western medicine. Even if the government is authorised, we are still not yet prescribing them, because we have limited knowledge on the efficacy of the medicines.

We are not yet educated well enough to start prescribing, but then we do have the knowledge on how it works. And we are also trying by all means to keep it that way. We are not trying to make drugs out of the traditional medicines.

We want to keep them. If there are roots, we try to give them as roots. If there are bugs, we give them as bugs.

So we are trying to keep it that way. That's what I think.

[Speaker 4]
Okay, so my view on traditional medicine in Zimbabwe is, I think it's present but not integrated. We are still far from that, because inasmuch as yes, we have people who practice traditional medicine, but I feel as if for the general public, it's not widely recognised. We are still far from that.

That's what I think.

[Speaker 1]

I also share the same sentiments with you. Like for myself, I was not a young man. You just hear it maybe on the media, but maybe not knowing whether it's something that is being done in terms of us doing it.

So it's not integrated as such. And considering also the community, they are working in the local environments, and with the Christian community, it's totally negative about this traditional medicine.

[Speaker 7]
It's so...

[Speaker 2]
So here is some kind of mix that we have that is more, I guess, but there is maybe some utilitarian, you said modernised, and when we see that in the system there, some of you said maybe there is some, at least on the policy side of inclusion, but how does it sit in that? Do the rest of you agree with that? That there is some kind of attempts to be inclusive on the policy side?

[Speaker 5]
I think on paper, yes, but then practically we haven't started practising it at all. Because I think we only have a few, few, few traditional pharmacists out there, or even practitioners that are really, really authorised to practise their stuff. So I think on paper it's there, but then we haven't started practising.

[Speaker 2]
Is it heading in a direction here, or has it been the same for a long time? Or is it progressing somewhere?

[Speaker 5]
It's progressing, but slow, slowly.

[Speaker 3]
Now I think the challenge is most of us don't actually accept the scientific part of the traditional medicine. We think that it's more the spiritual aspect rather than the science. So now because of differences in beliefs, like this one says, no, I'm purely a Christian and these traditional things, they are in tears, they will end up saying, no, this bullshit is the good way.

But some are now accepting it and understanding that, no, if you're saying I'm suitable, it's just a process to have in a special way. So if I take the wrong path, sometimes it might also help in the same way that I've said in the book.

[Speaker 1]
And also I think the community as well is also moving towards traditional medicine, in the sense that previously there were no groups whereby people would talk, maybe on social media about traditional medicine, about herbs and things like that. But you find that now there's so many groups and there's so many people who are even advising each other, what to take some, but don't just say, no, I've got this condition, what should I use it? So I think the community as well is moving towards that, though the challenge comes to what you're saying.

[Speaker 4]
Yes, and we can separate the two, the spiritual part of it and the scientific part of it. Because I definitely agree with that. There's some form of lack of knowledge when it comes to traditional medicine, its uses.

People doubt, they think it has no therapeutic effect as compared to scientific medicine. So they would rather stick to what they know than start from the beginning, and that's why I agree as well.

[Speaker 2]
But if we don't think about the statistics related to the WHO that claims that on the African continent, 80% of the population at some stage seeks traditional medical practitioners. And access to traditional medical practitioners, especially I guess in rural areas outside major medical centers, might be just a hundred times better. 200 versus 20,000 to one.

So it's a lot, 200 times more available, or a hundred times more available. So the question then becomes, what do you make of those numbers and that statistic?

[Speaker 5]
In Zimbabwe it could be, I don't think it would be 80%, but then we have that religious sect, the apostles, those that wear white, they hardly come to seek medical attention at hospitals and everything. They are big on their own traditional treatments and it works for them. So I would say yes, and people in rural areas, there are different religions, tribes, you know, advocating and administering their medicines.

But then I think we are still to go and learn more from them and see what they also see from those, or even test out their medication if they work. But then, yeah, it's true, to some extent.

[Speaker 2]
Because it's something that Abrejo emphasized, this question of accessibility. Yes. To medical, some kind of medical care or healthcare.

[Speaker 3]
And also, I also think the other part is, people tend to question whether traditional medicine is actually a permanent solution. For example, I have met two patients with breast cancer, and at the early stage of their cancers, they tried using the traditional way. They used to use that.

Guru used that in Shambhali, where he said to cure cancer. So what happens is, during that window, the initial stages, patients, they tend to get better. But two, three years down the line, it just comes back and it will be more aggressive.

So now people tend to question whether this thing actually works, or it's just a scam of some sort, which goes back to the point of lack of knowledge. We don't really actually know whether those who have these sufferings, the malignancy, in a certain window of time, or it actually does something to it.

[Speaker 5]

I think if we studied more, they could work hand in hand with the conventional tablets that we have already. Because it works to some. For some, they say it's gone.

For some, it will come back after years. So we have to study more. That means we have to be open-minded even more to accept that there's something else other than just western medicines.

[Speaker 4]
I think the reason why most patients might lose faith in traditional medicine is what he was talking about. We have a situation whereby I'm a traditional healer. And I tell you definitely, a hundred percent, this will eradicate whatever disease that you have.

I've given that assurance to the patient that I know you're not going to be sick anymore. You'll be fine. And then the next thing, I'm okay for a few months.

And then two, three years down the line, I'm sick again. So it's that, okay, so the patient is not sure whether this truly works. And also, I'm sure the practitioner doesn't really know if this truly works.

So I think it's a matter of actually wanting to educate yourself, you as the practitioner, and you as the patient, to see whether this is effective enough. And if this offers a therapeutic effect that's better, then yeah.

[Speaker 2]
Okay, great. So if we move down further, we have three. I just want to take some minutes to think about myself.

We've now seen that we've talked about the role of traditional medicine, surely. But we have this kind of, there's a highlight of this kind of one health approach that comes from WHO that emphasizes this kind of the whole health of human being in a health environment without the health of beings. And the role that WHO themselves highlights the kind of, it's the sympathy between this kind of one health approach and traditional medical approach.

Thinking about, and that's, of course, what's come through in a lot of our discussions during the workshops, of course, to address a human specifically, which is quite a complex challenge which includes people's behaviors, the environment they live in, their general health states, and so on. It's much, to some degree, why did it have specific use, a specific infection, or a specific disease, but a societal process. So we understand that traditional medicine sits there.

So I want you to think a little bit now yourself, all right, on these three questions. What role do you think traditional medicine has played in working with AMR, especially around the AMR education? Has it had a role, and how that role could, what could it look like?

Secondly, what are the challenges around integrating traditional and conventional medicine together in the AMR education? Then we want you to draw on the discussions we had during these workshops around AMR as this kind of question of, of course, following up, completing courses like they are in the audience, but also having a strong health state to

avoid getting infected. And finally, how could a conventional medical profession and a traditional medical profession get to work together around AMR challenges?

So those are the three questions I want you to take a few minutes to make notes on, and then we can have a joint discussion around this. Okay? Okay.

Let's give it two more minutes. Okay. Let's get on with it.

Okay. Let's see. We'll start with the first question then.

It's a, to me it's a very interesting question to hear your thoughts about that. What role has traditional medicine played in arresting the minds of all human beings?

[Speaker 3]
So, first question, I think it has played quite a role without people actually paying attention to it because you find out that in traditional medicine someone has a knowledge about a certain herb or a certain form of remedy. They try to keep it within their community. For them, they're always saying it's because our ancestors have given this to us.

And also, the knowledge of which herb to use for which circumstances is confined to one person, which means that there's sort of some control on the use of those herbs so that there won't be much of abuse of the herbs. And also, I think most traditional health, there are more immune systems rather than targeting specific parts, like the alveolar immune system, which is actually more needed to fight the existing infection or disease.

[Speaker 5]
Well, I think addressing AMR in Zimbabwe, the traditional medicine has played a major role because when someone is prescribed to start taking their antibiotics, let's say they have a flu and their antibiotic is slow acting, it has to take them seven days, probably on day two they're not feeling any better. They might just stop taking their medicines and switch to the traditional medicines for a quick fix. So I feel like it has played a role, and to those that decide to take their full course and as well as take the traditional medicines, we really don't know the drug to help interactions that we have because we haven't really tested them out.

We just know if you take your guava leaves and your lemon and what was, you mix them, you take them, they will help you ease your cough and everything, as well as you're taking your antibiotics. So maybe it will be adding, we don't know. I can't say it was adding to it or maybe it was helping, but then it has some effect on AMR that we are having here in Zimbabwe.

[Speaker 1]
Yeah, I also think traditional medicine has played a part in addressing AMR in the sense that maybe if you look at yesteryears, according to what we hear from our parents, it was normal prevention. You'll be given medication to stop you from getting this, you'll be given what you need to stop you from getting this. And also the fact that traditional medicine, no matter if I say I've got a headache or if I say I've got this, you are given that medication maybe once or twice, unlike the conventional whereby I'm told that I'm supposed to be taking my anti-hypertensive treatment for life.

So with traditional medicine, it's maybe when you have the treatment, they give you something and it's for a specific period of time. And if it's something that even my husband can't take, he's going to give me the type of treatment that he's given me.

[Speaker 2]
And now with the anti-hypertensive treatment, I was able to use the natural things like grains, you know.

[Speaker 1]
So I think it is great.

[Speaker 2]
Thank you. We were just talking about the first question then, what role traditional medicine has played in addressing the war in Zimbabwe.

[Speaker 4]
Okay, I think traditional medicine, it does offer an effect to the body, I guess, that will assist, let's say I might be taking antibiotics or anything whatsoever. If I'm more boosted and if I'm taking a certain part of traditional medicine, I feel like it will assist me to an extent whereby I might be able to fight that resistance.

[Speaker 2]
So it's preventive. Yes. Sure.

Sure. So then moving forward, what do you see as the opportunities and challenges around, we now talk about integration for AMR education, for engaging with these questions about AMR. I think like what Sasha and Doc said, it's a great opportunity if we bring the two together, if we consider the traditional medicine as a preventive, let's say boost your immunity, and then you take your antibiotics.

[Speaker 5]
I think it would work more, but that is if we are to educate people on the importance of sticking to antibiotics as well as sticking to your traditional medicines, not taking either or and just changing. So I think it's a great opportunity if we bring the two together.

[Speaker 2]
Do you see other opportunities? I think the opportunities, they're there, but it is a case of sharing the knowledge.

[Speaker 3]
Let's say for example, the most common thing, up to today, no one really knows how someone would expect.

[Speaker 5]
Yeah.

[Speaker 3]
Everyone is using it.

[Speaker 5]

Yeah.

[Speaker 3]
Two years back and there are some other things as well. So I think it's actually an opportunity to anyone on how does this thing work. Does it have any other effects that we are just negating?

Is it actually more helpful? Is there another use for it? And also, I think that all these traditional herbs, if there are some records being made by different centres, different hospitals, they say this type of a tree, they describe the component of the plant which they are using for a specific condition or something which they have noticed in humans.

Let's say it could be a running stomach or something. Or maybe, let's say, in other areas, especially rural areas, they say that if a lady is going for the first time, if she uses cow dung or elephant fecal matter, it will be better for them. Like the medical process will be easier for them.

And they won't be sustained to as much complications as the ones we can choose that. So if we try to find out what is it in those cow dung and elephant waste, I think it will be more useful. And it's actually a window of opportunity to integrate the two.

[Speaker 6]
Any more thoughts on the opportunities and challenges around the integration?

[Speaker 1]
I think the chances are there. But because this is very, maybe, enlightening. Because I think people come here by people are actually worried about the side effects of medicinal medicine and the toxicity.

They don't know the toxicity levels. They don't know what are the side effects and the dosage. Especially the issue of dosage.

So if maybe people are enlightened on that, it becomes an opportunity such that people have a choice to say, no, I'd rather go and see him or I'd rather go and see him. And then one challenge is I think, I was looking at it this way, that I don't know, maybe, I'm not aware of this. There is no school that will say people go and learn traditional medicine.

I don't know. Maybe it's there. So now people wonder, what I'm getting, is it the correct thing?

Is it the right thing? So if there were maybe traditional pharmacists, maybe people being trained in traditional medicine, then I think it would address those challenges. But as long as people are not aware, it becomes a challenge because I cannot go to him.

Because I don't know how well he is with the traditional medicine. Is he giving me the right drug? Is he giving me, I mean, the right dosage?

All those things. So those are some of the challenges, that as long as there are no, maybe there are no schools to train those people. And then also the norms and beliefs.

[Speaker 4]
I also agree with that. We live in a science-based world and look at the question whereby opportunities and challenges, when it comes to integrating traditional and conventional, we have more challenges than the opportunities. So as long as we don't counteract on the challenges, we're going to have a list of the opportunities.

But mostly with the challenges it has to do with people, if they're the studies, the studies are enough. And is this help in studying enough for it to be given to a patient? So those are the major challenges.

Also it has to do with dosages. Is this effective enough? And is this the right you have to take for this treatment?

And so on.

[Speaker 6]
We're thinking about the third question then.

[Speaker 3]
And how could conventional medical practitioners, conventional and traditional medical practitioners plan to work together around, especially the emotional, if we know that the question may Well that part I think, working together, not despising each other, not despising each other's point of views, trying to understand other people's point of views, and try to make it seem better. If you have found or someone claims that this treatment is good or in such and such a condition, try to find the evidence, or try to dismiss it with evidence, rather than just saying you don't have evidence, hence it doesn't work. We should sample and share the evidence.

Because there is lack of documented evidence for the use of traditional medicine. The conventional medical practitioners, they tend to say it doesn't work, it doesn't And also the traditional medicine practitioners, some of them they tend to say antibiotics, you tend to have these and that side effects, they are not meant for our bodies as Africans or in such and such a tribe, so hence it doesn't work for us. So trying to understand how their point of view affects you, and how to integrate their view with your view, that way I think it will be much better.

[Speaker 4]
So I also think that conventional medical practitioners and traditional medical practitioners could work together if they understand that their one goal, and that is to fight off resistance and its challenges. So if they work along those lines, knowing that we do need each other, the conventional part of it, the traditional part of it, indefinitely we could. So that means also getting ideas from the traditional practitioners, and also working hand-in-hand, understanding and educating each other on all sides, and also in order for us to be able to educate the patients on the use of both conventional and traditional.

[Speaker 1]
I still go back to what I said earlier on, that maybe I think yes it would work, in the sense that they respect each other. But I think for this to work, maybe there should be a school of some sort, so that these people are maybe at the same wavelength. Because imagine, he is,

he is, he is, he has done a level, he has gone through the five years of training, and maybe I just did grade three.

And for me and you to interact at times, it becomes very difficult. So as long as maybe there are people who focus in traditional medicine, and maybe there are schools for people to go through such, and maybe they can always maybe meet with the ones who are studying conventional medicine, and those studying traditional medicine, and then share ideas, and then maybe later on. But like how things are right now, I think it becomes a challenge.

Because everyone wants to know, is this medicine able to treat cancer, as you are saying. So like now, from him you can say that people are going somewhere, they get better maybe for a year, they come back. So eventually what will be his, I mean, you only think that no, this doesn't work.

But maybe there is interaction, maybe in the schools, then people will know. And maybe there will be trials as well, because with medicine there are trials. People are not just given, even antibiotics, when they are, when they are, when they produce antibiotics, they do trials, they do all sorts of things.

But with traditional medicine there is nothing like that. Somebody is just saying, I can treat. And it's saying, how far can you treat?

So, thank you.

[Speaker 2]
Thank you. So then, you see that there are a couple of things that are highlighted, at least in the research I've been following, is that traditional medical practitioners are, in the research often, presented as a potential connection between communities in healthcare and nursing care. And this of course bears out quite recently, based on the WHO numbers, that you have a much more saturated presence of traditional medical practitioners in communities versus conventional medical practitioners.

So, two aspects that I thought was interesting to highlight from the research that I was reading up on was, the first was of course the multi-health stakeholders in the network. And again, like I said, of course we've had, in our experience with COVID of course, but of course the recent outbreaks around cholera, we've had it here in Zim, and also news here in Nevada about that previously this year. We've had it of course in Amsterdam, in South Africa.

We've had a quite a bit of cholera response here. So traditional medical practitioners can be completely effective in monitoring these outbreaks, and there's a very direct effect on them. They live often, they live in communities, and are likely to be the first to work in any of these services.

So, open lines of communication between traditional medical practitioners and community of conventional medical medicine could tremendously improve surveillance, as argued by the research that I was reading up on. That health officials must then include traditional medicine medical practitioners in the education outreach. Again, we have to come back to

A1 education, and doctors , conventional medical practitioners, and trying to know what information they should request from traditional medical practitioners.

So it's this question of interaction that I'm going to touch on. So traditional medical practitioners could then potentially, as I said previously, be told why, what, when, and how to report unusual symptoms in patients in the health system at large. Again, this is counter-intuitive information.

The suggestion that you're introducing maybe checklists or pictorial guidelines, that's what Victor was highlighting, that you might have different educational backgrounds. But then the research has been thinking about that, highlighting, for example, you have the pictorial diagrams or checklists for symptoms, diseases, and modes of transmission, facilitating communication between traditional medical practitioners and conventional medical practitioners. So that's one side of it.

The other side that I find interesting is the question that comes up often, of course, around AR and microbials in the buildings, is the compliance. And continuity of compliance and behavioral change. So again, patients, at least if they are using traditional medical practitioners and visiting these organizations for research, it is the well-being that gives them the potential to make a big impact.

Patient compliance and partnership, collaboration from, for example, a conventional medicine by medical team. So this question of being in the community, being close to the patient has, of course, a potential, at least. And reaching patients maybe with this or without having access to a conventional medicine in the health system is also an interesting area.

And examples that I could find, for example, as a course with TB, is that if you have patients with dementia, with TB, as you know, it is a long-lasting hospital, but it is treatable. But the challenge then is the six to eight months of continuous taking of antibiotics reminds me that people drop out, whether a lot of economic reasons, or they don't see enough improvement, or whatever reason it is. There's multiple reasons.

So I found one study, it's not from the museum. I was looking for studies from the museum, but I don't know if it's being provided. But a study from South Africa, New Zealand, found that being 9% of those supervising a traditional medical practitioners in that study are completely legitimate, compared to 67% supervising other volunteers in the community.

And also, in the same study, the death rate of traditional medical practitioners and patients was two-thirds lower. Of course, there was good cases given antibiotics, but this kind of compliance seemed, at least in that study, to have been very powerful. Okay.

So what I want you to think about a little bit now is this. So if that is, maybe we can start with your reflections first of all. What do you think about those two aspects?

What is your immediate... Is it something you consider to be touching a little bit on in terms of the previous discussion? What is your view or your thoughts about those two aspects of how traditional medical practitioners can be important in a specially remote area?

[Speaker 3]
So I think actually the traditional practice is actually very important considering that most of our population, especially young people, they tend to vote for it first, mostly because that's what they can afford and that's what's available to them. But at the same time, if they can afford vote, they should be someone who is able to tell more on the effects of using both and not using both as much as they should. Like, if they drop out from the use of antibiotics and just say, now I only have the money for the antibiotics for the full course, they will not switch to the traditional use.

So at the end, it means that they should be someone who has knowledge at least in both the side of medicine.

[Speaker 1]
I think that the medical practitioners and those people who are doing the traditional medicine, I can see maybe the community, looking at the rural areas, communities are already on board. Because when people do integrated disease surveillance, they are being told maybe to look out for any animals that would be dying, maybe people who are experiencing certain disease and things like that, and they are reporting, yes, yes, yes, yes, yes, the traditional medical practitioners. But when it comes to the cities now, whereby maybe we've isolated the traditional medical people, it's a challenge because in cities, that's where most people are practicing Christianity or Christians, whereby they are shining.

And for one way to go there, I have to go there during the night. So that maybe if I'm a Christian, I'm saying, no, you shouldn't see me going there. Because, so, this issue of education, I think it plays a part when people are educated.

Then, like I've said, the rural communities, they are already on board, yes. They interact. But also the challenges maybe within, in terms of policy as well, maybe it should be policy because for me as a nurse, to give her all the education and say, no, you can go to school, you can do this, you can do that, it's not within my scope of practice.

So, I think there's a lot that needs to be done. But, yes, medical practitioners, if they rely on the, I mean, the traditional medical practitioners looking at how they practice, like I said, they can just give you one dose for maybe the illness that you are having or the disease that you are having, or maybe two doses. Unlike the conventional medicine where I have to take that drug for life, like we are talking about TB, six months, maybe the traditional medicine practitioner will just say, no, you can take this for your cough for seven days.

And I would vote for that.

[Speaker 4]
Okay. I just wanted to say that the open communication line is a need on both sides, those practicing traditional and those practicing conventional. That was highlighted that traditional medical practitioners have to be taught when to give the patient, what to give, what dosage, and is it the right patient, and they have to report unusual symptoms.

Because if you really check the way they operate, they really don't do a certain follow-up on a certain patient. To them, it's, I've given you, and I'm sure, 100% sure that it's going to get

rid of the disease and you're going to get better. So there's need for that, that open communication with also the conventional doctors.

So they need to be taught, they need to be educated, and also they need to learn that as you were saying, they say for someone who is taking antibiotics, it's a full course in order for you to get better. It's something that you can't leave in the middle because you're trying as much to avoid it, to fight off illness. So, and for someone who then goes, let's say, to a traditional medical practitioner, they'll tell you maybe that you don't need to take a full course in order for you to get better or something like that.

So there's a need for open communication on both sides, and as I was saying, education, I think that's key, that has to be the number one factor on both sides.

[Speaker 1]
And also maybe to get, like you were saying, that they shouldn't leave it on up on the shelf. What's the time to find out that you go to a traditional medical practitioner, you will tell you, no, stop the antibiotics or stop the anti-hypertensives that you are being given. It doesn't work.

Stick to what I'm giving you. So I think there's need for open lines of communication whereby they respect each other's views. And maybe the traditional medical practitioners, like she has said, have it such that people will know that when I have a headache, I'm going to be given this route, and for how long, and it's going to, maybe, the challenge comes now when we don't know the percentage.

And they also need that need to follow up as well. Was the patient treated? Because maybe what treats him is not what treats me.

Just like with anti-hypertensive medication, you find that somebody is taking enalapril, somebody is taking something else because of maybe the side effects that one will experience. But with the traditional medical practitioner now, it's just like what they say, one size fits all. That medication that is given to me is the same medication that will be given to you.

So I think we have to communicate with each other and then educate each other on why we do give this. And I think the effect of the lab also should play a part.

[Speaker 2]
We've touched a little bit on that, this kind of misuse of enalapril, and what are the necessary terms of collaboration between the traditional medical practitioners. So we've touched about that, of course, communication, avoiding animosity, I guess, is necessary. But what would we say are the necessary terms?

What would be necessary, especially when our engagement is largely a question of not just medication-specific disease, but the larger challenge we're facing. What conditions need to be in place to enable that kind of collaboration? Because we say that, okay, we need communication, we need respect or understanding, but how do we get that?

What would need to be in place?

[Speaker 3]
So on that part of AMR education, I think as a person, as a patient, we have a right to choose the model of treatment we should want. By so doing, us as practitioners, we should know better in our field. As a doctor, I should know that if I prescribe someone a prescription for seven days or so, by day four, I should be expecting some changes.

The patient should be getting better. If they are not getting better, they should come back to me and question myself. I should be able to question myself, am I actually doing something?

Before that patient just takes the measures into their own hands and say, apart from this machine, let's go to another machine, which would be maybe the traditional way of taking another form of antibiotic on their own. So yeah, if you show confidence in your knowledge, and if you have the knowledge of that form of medicine, whether it's traditional or it's the conventional way, people will trust you and will trust your ways. And if they work, you have to also try to, especially with the traditional medicine, try to make some documentation of some sort, so that there will be evidence that such a person, this group of people, they were treated using this way or this habit, and this was the outcome.

And that will also be used in the nearest future to treat the same other conditions with such types of herbs or medication.

[Speaker 4]
Okay, so I think anything that has to do with a patient in regards to the form of medication that they take, whatever treatment that they receive, there have to be policies in place. And from there, it becomes easier to know, it will be easier for a traditional practitioner to know what they are required to do. At the same time, for a conventional medical practitioner to know what they are required to do in terms of treating the patient, in terms of what you're supposed to give the patient.

So policies are very important for that collaboration to work. So from policies, we then have to move maybe to guidelines as well. You are supposed to be guided, the both of you, so how are we going to treat those things if we are to set in guidelines?

There's need for policies, yes.

[Speaker 1]
And also, there is need for evidence-based practice. Because as long as there is no evidence-based practice, then it becomes difficult. Whereby we are saying, you know, this drug, we are going to have this drug which is going to totally treat HIV, and people want to know, are there any studies that have been done?

So, yes, in traditional medicine, as I said earlier on, they might also give a once-off dose, but maybe the side effects are very bad. So I think there is need for them to work together, and then maybe if there is evidence of what they're treating and how they're being treated, then I think it also helps in reducing antimicrobial resistance education, because one will know that, you know. So and so went to say Uganda was given this to treat cancer, maybe for two weeks or so, and maybe one would propose to say, no, I think I would rather go to the

traditional medical practitioner or to the conventional medical practitioner, looking at maybe how long one is there.

Because that's where the challenge comes, no matter. Because even if the four of us were going to ask ourselves who has completed the course of antibiotic, maybe observing the time and whatever, find out, maybe I'm going to take that drug that I was supposed to take maybe for seven days, maybe still taking it on the end. Or somebody stops, because one would just know that, no, I'm not being treated.

So I think as long as people are aware of the importance, maybe, of giving medication, whether it's a traditional medical practitioner or the conventional medical practitioner, in any form for a medical practitioner, maybe looking at the patient, maybe asking questions like, have you ever taken an antibiotic? And maybe before that, even looking at prevention, before we even think of giving those antibiotics or those medications.

[Speaker 2]
So just to wrap up, then, we have two questions we really want to touch on before we end today. The first one is this question about how we've talked a lot about the traditional medical practitioner. The question is also, communities are their own schools.

So how can communities support an integrated whole system of prevention, of intervention, or what can communities do to support this kind of integration?

[Speaker 4]
OK, so when you're involving the community, you have to get stages to it. So the first thing, maybe we can start by gathering the community, having meetings. Get to know your community, introduce to them whatever that is.

So those regular meetings, educating them on that. Then, upon that, you can then talk about also involving the traditional practitioners and the conventional practitioners to then come talk to them about it and educate them more. Because the community, they need to hear from the practitioners.

Then maybe from there, you can also have health care workers from the community that can work hand-in-hand with the practitioners. They can assist each other. Yeah.

[Speaker 1]
I think that if people are enlightened, as I was saying that in the rural areas, the communities, they support the traditional medicine. But if you come to cities, people tend to shine. So if there is policy, people are enlightened, then it becomes easy to intervene in this.

Because people will know that this is something that has been approved. There is policy that I can make my own choice whether I'm supposed to go to the traditional medical practitioner or to the conventional medical practitioner. Then, I saw that they can address the issue.

Because as long as it's focused, larger populations are in the cities. Smaller populations are in the cities. And not all of those people live in rural areas.

But maybe three-quarters do. We live in traditional medicine. But when you come to cities now, that's why people are saying, no, I'm a Christian.

I don't believe in it. So the most important thing is for people to be enlightened, to be educated. And then policies to be put in place means you can address the issues of the traditional medicine.

[Speaker 2]
So if we finish up then with the last question is around how could an integrated health system that we talked about throughout the conversation today, how could another country support community health as a whole? This kind of holistic health in addressing it. Whereas if we might have seen a lot of challenges of achieving this integration, so what are we striving for?

What are the visions now? Because if we're going to try to overcome these challenges, it needs to be, of course, a worthwhile pursuit. So what do we see as the kind of goal there if we are going to try to work for an integrated, more integrated health system?

[Speaker 3]
So for the third question, I think as modern medicine we have to gently introduce the scientific part of our view to the traditional medicine without trying to shun their spiritual aspect of their medicine. Because it seems with traditional medicine what they believe is this bush works because of our ancestors or because our spiritual beings, they are allowing it to work. So if we try to understand their point of view and then we try to work out the scientific part of it without actually dismissing their point of view, that way we may get to answers of how certain traditional medications work and they might actually open up more to these different kinds of herbs which they use.

And that way we might eventually try to address the antimicrobial resistance which comes as a result of ignorance of not knowing the effects of certain herbs on the human body.

[Speaker 1]
I would say maybe, if we look at it now, if we look at the conventional medical practitioners in educating patients with traditional foods. And with traditional foods, yesteryear when people used to eat traditional foods, then they used to have no challenges, sort of prevention. So now that's the teaching whereby people are told, no, don't drink this, just drink your water, don't drink this, don't drink this, this, this.

So in a way, conventional medical practitioners are actually educating people on that. And maybe if now they recognize it, then they can maybe, if they interpret it, then maybe they can look at the issues maybe of the drug, you know, whatever they give the patients that they give in the first place. And then just try to use that.

But as I look at it now, I find that we are way back there, whereby people used to say you have to do your whole brain and things like that, and like the process is things that we are doing now. So in a way, if people continue maybe to have that view and put the traditional medical practices as a policy, I think it's all right.

[Speaker 2]

So you would say that the traditional medical practitioners more often actively include the question of diet, food, ingredients. Yes. So there we have an interesting aspect, I think.

Because we touched on the question of food, lifestyle, those things throughout these workshops. And here we have something around like the holistic health. And as you said, the traditional medical practitioners are also there much more in the community, at least in rural areas, around like supporting that kind of order in healthy lifestyles.

Any final thoughts on what we should strive for regarding that topic? Any other final reflections on that? To go back to the theme of today's workshop, which was about health system integration for addressing evil, that we've been exploring the relationship between traditional medicine and conventional medicine in approaches to equal education.

What is your, like, final reflections on this?

[Speaker 4]
Okay. For me, I know we live in a world whereby we think, or rather, a greater percentage of people think that traditional healers, they should only be recognized, let's say they're not recognized as medical practitioners. But maybe what people don't know is they can also be integrated into the health system.

They do have their own policies. They do have their own teachings that we can also benefit from. So the purpose of this, of building an integrated health system, always goes back to education.

And first educating the practitioners themselves before they educate the communities. So yeah, those are the things.

[Speaker 2]
Any final thoughts?

[Speaker 3]
If we integrate the two, or measure the value of that, the two is one. If we accept that traditional medicine does exist, we try to measure it, and we get people to accept it as it is, and to find out more, and learn more on its effects and impacts on our daily basis, activities, and our health. And also, we will find out more discoveries, and I think it will make better outcomes in terms of our drug control as a whole.

[Speaker 1]
Thank you. I think this interaction between conventional medical practice and genetical practice is going to have a great impact in the ultimate of your existence, in the sense that maybe, as I said, people will be looking back at the type of diet that they used to do, the type of lifestyles. For example, I'll give an example of now, whereby I just get out of this building, I get into the car, I go home.

From home, I get into the car, I go somewhere else, which is quite different from the traditional component of it whereby people walk long distances, and they are actually kind to their hearts. And maybe, as we bring, using the elevator, you know, I shun the stairs, so

maybe, if these people interact, then they have an opportunity to show why, you know, to say, this is good at doing this, and this is good at doing that. It becomes very helpful.

And basically, with the traditional medicine, people are, it's more of a relative. People are doing things, they're eating something to prevent, and yes, it can reduce Thank you so much.

[Speaker 2]
Let's wrap it up. Thank you so much for your time. It was very valuable.

I think it's, for me, it was very interesting conversations. I hope you also found that you got something out of the discussions. We also wanted to share also with you, around the time of the workshop on Tuesday, next week, that we will be setting up, we're going to talk about connecting into the veterinary power, the mental power, and also do like a little bit of checking also how it relates to what is happening in your everyday practice, from the previous workshops and to this workshop and so on.

So, we tried to start it, we were still trying to run it by ourselves, but it's a difficult time, we said, it would be better if we adjust the times a bit, or what do you think would be viable for enabling people to join on Tuesday?

[Speaker 1]
I think people should be given enough notice, because the times should notice that people have already planned, like maybe other people have gone for exams, these are things that are already in place, and maybe if they tell us, maybe, I should say two weeks before, people can plan and say, no, we cannot come on this day. Maybe 500 people, 500 or so, can say, no, we cannot come on this day, because then we can reschedule for another day. But with this one, it was two weeks, some people had things in place for it.

[Speaker 2]
On Tuesday, it was a list, it was only two weeks. This is for last week.

[Speaker 4]
I think it was, we, she didn't know, but we already had plans, so. So WhatsApp is much better than email. Yes.

[Speaker 2]
So how does it look for you on Tuesday?

[Speaker 4]
For me, Tuesday I'm going to be ready.

[Speaker 2]
Do you have a plan for Tuesday?

[Speaker 3]
Yes.

[Speaker 2]

That's what I was wondering, because it's better that we're running in time, and one is for 9 o'clock.

[Speaker 4]
Yeah, I think 10, we're doing 10. I was just wondering if I could find some. It was Friday.

[Speaker 1]
I was just wondering if I could find some.

[Speaker 3]
And I also think, like, you know, there is the consent forms.

[Speaker 1]
There's something that's totally different with the consent form. And I also think, that's my thought, whereby there was an amount that was stipulated, but now people are not aware, is there a way to stick to that? You know what, some people, I can give an example of my son here, maybe he comes from somewhere far, then he has to use his vehicle to come here.

So you can imagine somebody driving from maybe, what should I say, the farthest place? Yeah, so those are some of the effects, because like last time people asked, and it wasn't clarified, but on the e-consent form you said we're going to get $40, but now nobody blamed it, so it could also be something where people would say, where should I go, I waste my petrol, I drive there, I have to fork out money from my pocket. Yes, we worked on that.

[Speaker 2]
So we managed to get the funds for that in the consent form, it's a reimbursement up to $40, and I think Mrs. Mussolini has been managing that papers, so those papers have been here, and we will be able to hold that next week for these two workshops. So yes, it was a bit of a, I had to work quite a lot with my family, but we managed to sort it out.

[Speaker 1]
I remember one of the senior doctors asking the other time, saying how do you expect us to travel and come? I think it's the second workshop, but they were not doing that. So it could also have effects, I'm just thinking.

But maybe people need to be notified on time. Find out that, like, people will know that we are coming on Tuesday. Some people will say no, definitely urgent, because there's ample time for people to plan.

That's my view, I don't know who are the others.

[Speaker 3]
And also there are some other challenges. Sometimes you don't get the permission.

[Speaker 1]
I was also thinking that since the clinical director is away, I don't know why the doctors would have challenges. I've told [redacted name] that she should talk to the clinical director, the personal assistant, so that they discuss these issues, because they have allowed them to

come. So I think it's just formally to tell them that today is the day of the workshop, and they should be excused.

[Speaker 3]
Yeah, actually they should make the formal recommendation so that other people can come.

[Speaker 1]
Yes.

[Speaker 3]
Yes, yes.

[Speaker 2]
Okay, thank you so much for today. I think there is enough for most of the time. And you were all on the list of Mrs. Mussolini was showing her up. Yes. She says that we will prepare the reimbursements for Tuesday. Thank you so much.

Harare workshop

[Speaker 7]
I still have a list of names, it's still in the... Yes, I saw, she sent a message.

[Speaker 2]
Okay, good. We can start here, it's the first discussion in one group and then we can speak.

[Speaker 5]
Okay, we can start here, it's the first discussion in one group and then we can speak.

[Speaker 1]
Okay, good.

[Speaker 2]
What is the relationship you view in ZIM around the relationship between conventional medical practice and tradition? Of course, these are like just a guide for the discussion, so let's...

[Speaker 1]
Okay.

[Speaker 6]
It's allowed. I think the relationship in ZIM, in view of the traditional and conventional medicine, it's trans-cultural, trans-disciplinary and to some point also sort of romantic, because the government is putting in place registration boards for people to practice both conventional and the traditional medicine. So it falls, yeah, maybe they've accepted the fact that the cultural part of it is still there even up to now, because you find it in the streets, people are selling, they don't even run away from the council, they have their barks and...

Roots. Roots and...

[Speaker 1]
The bulbs and whatever.

[Speaker 6]
Everything that should be like new, but it's there and they are selling and there are people asking, I want this for the others that have them, it's not like in powder form, they have written names but there is no dosage there is. But the government is allowed because we have Zinata, we have Zimbabwe traditional, what works in associations is there, so the government is... In Zimbabwe it's romantic and trans-cultural because that's my view.

[Speaker 1]
This is where we sit, isn't it? We are sitting on the trans-cultural and trans-disciplinary system, but I would want to go further to say the relationship, though it's very strange, those that are practicing the conventional medicine, they look down on those that are practicing traditional medicine, and the same, I mean the vice versa is true, to say the same as the traditional, they also look down on those that are practicing the conventional, because, you know, depending on why, maybe it's because of the religions, the different religions that we have, some don't even take any, the conventional or the traditional, they go the spiritual way, to say, I don't believe in medicine, I just take... My faith makes me...

[Speaker 3]
Sure. The trans-cultural as well as an integrated system, we do have people who are strongly inclined to the use of the traditional medicine, and sometimes very difficult to convince them. Even during the COVID era, the Mzumbani traditional roots and beliefs became so popular that even the health workers, those who were practicing the conventional, were even convinced that this is working more, it's more effective than the conventional.

So they used both, and sometimes even during the onset of HIV AIDS, there was a problem, a sort of a tug of war, they would use both, the traditional and the conventional. However, we also have a group of the members of the apostolic faith, the apostolic, they neither believe in traditional, the white garments, nor the conventional. They just depend on prayers.

We give you water, holy water, you know, and so forth. And to the extent that it's a problem, and even when we talk of diphtheria, what, what, they don't even believe in the immunization of kids. They are known for that.

However, the fact that, you know, they are pro-government, nothing is done to them. That's where they get their protection, their youth. So in Zimbabwe, there is the integration, the traditional, the conventional, and that spiritual aspect is very, very important.

And when it comes to the treatment of psychiatric patients, they either believe that, no, this is spiritual, they don't believe in these antipsychotic drugs, they all say it's spiritual. And people are forced and even convinced that, ah, no, let me visit them, until the problem remains unsolved. Even those who are drug addicts, they are still being sent to the traditional healers, and even the faith healers as well.

So it's a complicated issue. But however, the majority in Zimbabwe is conventional.

[Speaker 1]
Efforts are being made to help educate the community on respecting their culture or their religion. And like we said before, the government is making efforts to include the traditional leaders, the spiritual leaders, so that we incorporate everyone. No one is left behind.

[Speaker 3]
But recently I heard the Deputy Minister of Health, he was saying whether you are a traditional healer, a faith healer, you should have the two basic things, you should have a BP machine and a glucometer. Before you do anything, make sure you test for the blood sugar as well as the BP.

[Speaker 1]
But you know, [redacted name], we used to say, the testing of sugar was there even in the ancient ages, because they would monitor, they would maybe designate a place where one goes and urinates there. Then they monitor for the ants. There is a certain type of ants that comes and...

Which love sugar. They love sugar, so they would be on that area. When they see that, then they are sure to say, this one has diabetes.

[Speaker 5]
Back then, they used to... Before we used to test, there was a method that was used to test for diabetes. Some practitioners would even test.

[Speaker 3]
Yes, dip your finger, then you test. You actually...

[Speaker 5]
So the ants would be going for the sugar. If you put something sweet...

[Speaker 1]
My question is, if you use your tongue to taste, you actually... It tastes like salt and sugar. Ah, okay.

[Speaker 5]
Okay, on the first question, my view is, really, there are plans at national level to integrate conventional and traditional, but practically, I feel like they are more on the complement side. They just coexist mainly. Of course, there are plans.

Of course, we already have some councils, like you're saying, but at the moment, on the ground, for me, it's really some form of coexistence because the other side always is more... I'll use the word on the board, paternalistic towards the other one. It's just like that.

If we go, if it's the traditional side, they will always diss the conventional side most of the time. However, there are plans to integrate, and that's how I feel about it.

[Speaker 13]
Okay.

[Speaker 7]
I was thinking more of a co-evolution because complementary medicine has evolved. Traditional medicine has also evolved. We're seeing them now on the street, as Stepsire was saying, selling the drugs just there.

It's there. We don't know what constitutes what they are selling. Same goes for the conventional medicine side.

They've also evolved. We have people also just selling. We used to talk about this before, selling the drugs on the street.

Antibiotics and all that. But on the ground now, like what Dr. Kashi is saying, things are, they're there, they're there. But the integration part, I think that is where we really need to focus for the traditional healers to understand also what we're talking about AMR, for us also to understand what constitutes their drugs, their traditional medicines, what they're giving.

Because I had a case about three months ago. We admitted a patient. He had PVD and heart failure syndrome.

The PVD, we called the surgeons to come and consult and assist. They wanted to amputate. But his relatives now would come and put traditional medicine on the wounds that he had.

Concoctions. Yes, concoctions and powders, a gray powder. We were not aware until one day we opened the sheets, we're examining.

They're black things, black powder. Yet we're also giving him our medicine. You understand?

He deceased, he demised. So we don't even know from what. Because according to us, we were progressing and getting ready for amputation, for operation.

But ah, he just left us. It was a shock.

[Speaker 1]
It's the same as people in maternity. When they come to seek for the services in maternity, they don't actually tell us what they've taken prior to their visit. Then maybe now you are saying you want to augment.

You augment. Yet they have taken African Pitocin. Then we end up having a rupture.

And even if you ask them what did you take, they don't say. They don't share. They don't share.

But now when there is a complication, now there is a situation. When you ask, they will tell you, I have taken the elephant duck.

[Speaker 6]
It's an insect. So it lives in a house with mud. So they take that.

And it's very effective.

[Speaker 1]
When you see someone who has taken this traditional Pitocin, the labour, hey, it's, the progression is not like the natural, I don't know. Not psychological. We have seen it.

[Speaker 5]
Not psychological. It happens. It's not psychological.

It's not psychological. It's an elephant dung. It's scientifically documented.

[Speaker 1]
Usually when they are coming, they tie a stone. Once you take off that stone, the baby comes out. We have witnessed that.

[Speaker 6]
We always find this opportunity. Come on.

[Speaker 1]
We will show you the matombos and everything that they bring.

[Speaker 12]
I'm not sure in the Jordan culture, but in the Arab culture, someone is having mumps. So they take this cup of maize. You tie it around what?

[Speaker 1]
Around the neck.

[Speaker 12]
So when I was a kid, I used to think, this thing is working. But then I started to understand that, no, mumps is a, mumps is self-limiting. It can be very, but you think that, that thing which they tie it, is the one that's working.

[Speaker 1]
No, but they were not just tying the, the, the, the, the cob alone. They would even monitor the diet. The diet that they give you, they won't, they won't give you something with salt, something with, they've got the other restrictive diet that they would use in conjunction with the, with the cob.

And it has to be, it has been proven that it was working. I don't know. It depends with, I think it all starts with the socialization.

How you were socialized. You were made to believe in certain things, and to you, they do work. They do work.

[Speaker 2]
So, we've seen here that, that it sits somewhere in between, I guess, the co-evolution, the trans-culture, the complementary, the paternalistic. There is quite a complex situation. And it's also, it could maybe be sometimes romanticized, sometimes integrated, but quite often it also becomes a, maybe it's inclusive, but it's more tolerant, but it's, so it highlights this kind of question that it's often where you are, it changes quite a lot.

So, we were just, we were just discussing like the relationship within the Zimbabwean health system between conventional medicine, so where we are now, and traditional medical practice. So, any thoughts you have initially before we move to the next topic? Like about the relationship between conventional and traditional medical practice, especially around like, is it an integrated system?

Is it inclusive? Or are they just separate and complementing each other?

[Speaker 13]
Complementing each other. Ah, okay.

[Speaker 4]
I don't need to lie, and I don't catch up quite yet, but I don't think we complement each other. For example, the traditional practice in Zimbabwe, let's say you have an open wound, that includes traditional medicine, and it involves more infection and sepsis on the patient's wound, then the doctor is telling them, no, don't do this, you should come for dressings, daily dressings and things like that, but the traditional person will be saying, no, this infection works for you. So, I don't think there's an integration there.

[Speaker 2]
Thank you. Then let's move to the next. Now that we are a bit more, now we are seven, so let's split the group now.

[Speaker 7]
Are we doing the second question now?

[Speaker 2]
We can...

[Speaker 7]
I just want to add something.

[Speaker 2]
Okay, please add that.

[Speaker 7]
I wanted to say, conventional health practitioners are aware of traditional practitioners. Because even when we're clerking, we ask, are there any other medicines that you're taking? Not even just conventional ones, but also traditional ones, we do ask.

So we are aware of their existence, we do respect them, but we just don't want the, what do you call this, the intermingling of the medications, because we don't know how they're going to react.

[Speaker 13]
Interactions.

[Speaker 7]
Yes, interactions, that's what we are afraid of, drug-drug interactions.

[Speaker 5]
Great, thank you. Maybe on top of what she said, really, the traditional ones, from their point of view, is conventional. I would say somehow, they affect our management to patients.

Really? Because sometimes our patients present late because of some of the ways we're taken by the traditional, let's say. Due probably to lack of standardization by those who practice the traditional approach.

So they'll be maybe doing underdosing, and sometimes they'll even do overdosing. Since it's spread mainly by oral tradition, it could even be a wrong treatment, because it's really oral tradition, it's not quite documented. Unless if herbalists are going to, there's a council and herbalists trying to do something about it towards standardization.

Thank you.

[Speaker 2]
So let's move forward. Sorry, could you join this group? Yes.

Then we'll have three on four.

[Speaker 1]
Yay!

[Speaker 8]
Yay!

[Speaker 2]
I'm afraid that our discussion will begin to look at broadly the relationship of the conventional and the traditional medical practice. So let's try to make it a little bit more two-dimensional. Is that right?

There we go. There we go. So let's take just a while now so we can discuss it with the groups there.

These three questions. You can start with me. The first question is really what role does traditional medicine play in addressing MRT?

Does it play a role? And how does that role work? So it's a kind of a looking backwards, looking into the current state situation.

Because we talk a lot about MRT, about these workshops. This morning now we talked about MRT as being this question of holistic health. So can we imagine what role traditional medicine can have?

I think not. That's the first. The second one, what do you view as the opportunities and challenges around integrating traditional and conventional medicine, not generally, but for MRT, specifically for that point?

And thirdly, how can conventional medical practitioners and traditional medical practitioners better work together around this? So we have a little bit of looking at the current state, imagining what are the current opportunities and challenges, but also looking forward to what could we do better to better work together. Does that seem clear?

So please, you can take these questions in order and just discuss in your groups how you view these topics. You can start with the first one.

[Speaker 8]
What role has traditional medicine played in addressing MRT in Zimbabwe?

[Speaker 10]
Challenges. Role of traditional.

[Speaker 1]
But, do we have the microbiology in the medical tradition to make classification of drugs here?

[Speaker 4]
Like in antimicrobials and things?

[Speaker 1]
Because that's how we can only talk about, you see, Are they teaching them anything with regards to this?

[Speaker 10]
Yeah, I don't think they are teaching.

[Speaker 1]
What I know is maybe when they are given medicine, they are told to, like the instructions that we also give in the conventional medicine to say you take after you have eaten something or take before you have taken something or stay away from this whilst you're taking this. True. Those are the general information that they get.

[Speaker 4]
And when they see now that the condition of the patient is worse, let's say there's like an infection they can't control, that's when they'll be like go and see your doctor. They never give antibiotics. They don't know about them.

[Speaker 1]
Do they teach hygiene as well? They do. They do, but, you know, it's not as in the conventional practice.

[Speaker 4]
They're not as strict about that.

[Speaker 1]
So what are we saying now? What are we writing?

[Speaker 3]

Yeah, usually the traditional medicine was once you get treated there they don't normally keep files for you saying you were treated for this ailment two weeks ago, then you are coming back again with the same problem.

[Speaker 1]
They always repeat the same. I would want to differ from that. There was an issue in our family where we had to consult.

You know, we do have different backgrounds. So we called the whole family all together to say we need to go somewhere. When we went there, that's when we only noticed that some of us had gone and sneaked and went there.

Because when we got there, this traditional practitioner was like saying you were here last week and I even gave you this, this, this. Did you use it? You didn't use it.

What did you do with it? I think they are more spiritual people. Their reviews are not written down.

But they have, I don't know, they follow up in the spirit to say did they use this? If they did, is there any progress? Or there was something that you did amiss?

Like the instructions that you were given. You didn't follow them. You didn't follow them.

[Speaker 4]
I think there is a positive side to it that they do acknowledge their conventional medicine. Where they say, okay, we are treating you for, I don't know, whatever, is that they'll be using their traditional medicines. But like I mentioned earlier on, when they realize they can't control this sort of infection, they can't control, they'll not refer the patient to the government hospitals.

Then the other thing about traditional people, they can use, I don't know, is it firewood? Let's say it's a wound to burn, to stop, let's say. I know it's not something we promote.

But positively, they try to cure the progression of the condition, the disease, for example. Let's say it's a rotten leg, they'll try to, not to amputate, but something like that.

[Speaker 1]
I was looking at the Indians, the Indian people, they use herbs. Especially when one has a bruise, one has an ulcer or something, they use the turmeric, the turmeric whatever, the turmeric something, something. But one thing I was observing, they were like, they start cleaning the wound first, before they apply, meaning that they are also acknowledging that there is a thing which is called sepsis.

Though I'm saying we don't even know which one of their herbs do they call the antimicrobials. Because to them, we only know that they use herbs. But which ones are the antimicrobials and which ones are the, we don't know now.

So to say they have a stipulated, I mean they have a specific role that they have played with regards to a MR, how would we say?

[Speaker 4]
I'd say it's more to the conservative side, it's more to us, we are the ones who are worsening the resistance rather than the traditional people. And like what you said, we don't know which herbs has antimicrobial effects, but we all know that there's some drugs which are made from these herbs, that they dry them, then they put in capsules and then we now say, oh this is our medicines, but they were originally from the traditional side. So these people, they have the knowledge of knowing which one is the antimicrobial.

[Speaker 1]
Maybe if they could, maybe like when they're giving you these herbs of them, their herbs, they would maybe tell you this is more like an antibiotic. You need to use it consistently and finish the course. If you don't finish the course, maybe you are going to grow resistance or whatever.

I think that would maybe improve the practice that they are doing and maybe it would bring appreciation even to the community and the other practitioners. But because they don't have specific instructions to say these herbs, it becomes difficult. And it's not written.

[Speaker 4]
Sometimes it's very good to have a written document where your traditional doctor writes things for you. Because if it's word of mouth, I can now forget what you said I should do. But if there's an instruction, I can go back and read what did the doctor say, what are the instructions, how should I take this, things like that.

[Speaker 1]
But when I went to this level that we are talking about, they would give you like a sachet that is written for porridge, the name and where you are going to use it. Then they would tell you how much to put, like a quarter teaspoon, half a teaspoon. Though we don't know whether it's 500mg like you are using a capsule, but they would measure to say you use half a teaspoon, a quarter teaspoon.

In a cup, a 200ml or 300ml cup, you eat it before you take your meals. This is how I got so much interest. I wanted to know because it was detailed.

But most of us didn't use it.

[Speaker 4]
We just say, oh, it's spiritual. But there's the practical side where the herbs don't actually work for you.

[Speaker 1]
But my sister got healed. This wound had gone for years without healing. They did micro-culture insensitivity.

They did all sorts of trials. It wasn't healing. But when we went, it was soon after my father's death.

When we went there, she was picked from there. We are a very big family. She was picked from this family.

They said, you have a wound which is troubling you for some time. Just a powder, a very small powder. He said, you have to put it in the Vaseline because it's very painful.

But when you're applying, you need to clean your wound first. He said, use saline water. He said, go and check salt.

Put it into water, in clean water. Then you clean the wound. After you clean, then you apply the paste.

Then within three, four days, we could see the granulation. Then I said, so when he brought up the traditional, then I said, oh, OK. The medicines, they work, but the fact that maybe there is a need for further studies into these drugs, maybe to incorporate the scientists to come and see the type of drug and see what the properties of the herb may be.

That way it would come up with maybe something which is very effective. What are the opportunities and the challenges around integrating traditional medicine? I think during our discussion, we have already touched on the challenges that we are coming across, like those that we are saying we don't know the dosages and the properties that it becomes very difficult, especially when you want to apply to a doctor.

A doctor has gone to seek help from this traditional healer. You are given, you start to wonder, what's this? What is this?

Maybe if they could tell us that in this powder, there is a proportion of this, there is a proportion of such, such, such, such, such, such herbs. And when you try and relate it to the medical, the conventional drugs, you would acknowledge, to say, yeah, maybe I think it's safe to use. But because there is no composition of what makes the types of herbs that they use, it becomes difficult.

And a lot of people wouldn't want to use them because they don't even tell you the side effects.

[Speaker 3]
Yeah. Yeah, I think some of these, the drugs, yes, they are being classified. They can say this one is for treating STI.

This one is for treating cough. This one is for treating wounds. And these drugs are for treating dysfunction, ED.

They are good at that. They have these medicines. But what it contains, Havana, Rosio, if you say I am complaining of this and that, from your presenting complaints, they say, okay, you need this one.

Because when you get to town, there are so many bottles with medicine, traditional medicine there. But once you say, I've got a headache, you need this one. I've got a discharge, oh, okay, STI, you need this one.

You know, and so forth. And again, there are so many prophets going around. I come to you, this one is the best.

I am given medication. I consult you and I know the best one. You know, you are given.

Then you mix the two and there is drug-drug interaction. And so forth. People are bound to go mixer-mixer.

Because of, you know, as I have alluded to. So, Havana will be very difficult by education. You don't know what resistance is coming from your drug, your unfair truth or this one.

[Speaker 1]
But what are the opportunities there?

[Speaker 4]
What opportunities are on the table? I think opportunities will be a lot. Considering, let's see, now you are covering all the people.

There will be like the spiritual side of things, the traditional side of things, the confessional side of things. If we can integrate and work together, then we are giving the person not just the physical healing, but also the spiritual aspect of things. Those are people, they are really deeply rooted in spirituality.

So, just like how doctors consult, go to the cardiologist, go to the what. After you have finished with your examination and your treatment, you can say, I also consult, go to this traditional thing. Then they can do whatever you need.

[Speaker 1]
You know what, it seems I have testimonies on everything. On my second born, now he is 22. I delivered him.

He was more like a near term baby. But when he was born, the APGA score was 5-7. Then 7.

So the pediatrician was [redacted name]. You know Dr. [redacted name]. Then he was in severe respiratory distress.

So he was in the ICU, Belvedere ICU. [redacted name] was like coming. I stayed there about two weeks.

On my second week, he came and said to me, [redacted name], do you see the mother? I said, yes. He said, she is in the hospital.

Do you see the mother? I said, yes. Because this is the seventh day Adventist.

I said, today, I have the cards. Even the dose. And he even came on the seventh day.

[Speaker 4]
They usually do that.

[Speaker 1]
Yes. My sisters were saying, Ah, Dr. [redacted name] came on Saturday. Then that's when he made that confession.

I got a spiritual revelation today. I was in the hospital for the second day. I received the second dose.

I stayed there for a while. Then I was in the hospital for some time. I was in severe distress.

Then, third day, fourth day, I had an observation. Did we really achieve what we wanted? You know what he did?

He said, I want to see this son of yours frequently. I really want to mourn it. It's a miracle baby.

I wanted to second or to concur with what you were saying, to say if that could be possible to know what my chances are. Because according to you a healthy person has to be emotionally, physically and socially well. And spiritual.

That's what I was asking. Do you see a healthy person? Do you see a healthy person?

[Speaker 8]
True.

[Speaker 3]
That's why most of the hospitals right now are chaplains. There are many chaplains. They don't have hospitals.

Every hospital has a chaplain. Even at the parallel that spiritual aspect is very important. I remember during the old days Bishop [redacted name] was our bishop used to say there was a research which was done.

This is a group of hospitalized patients. No one would pray for them. This is a group of hospitalized patients.

Every morning they receive prayers. Those who received prayers there was a very good prognosis compared to these ones. Because that spiritual aspect if you get into stress if you are stressed your immunity goes down.

[Speaker 1]
Same as social. If there are no visitors to see the patients you get stressed. You get stressed and you get bipolar.

It's very vital. It's very necessary.

[Speaker 12]
It's needed.

[Speaker 1]
Then the third one is how could Medical Practitioners and Traditional Medical Practitioners better work together around a AMR challenge. I think it's not about the ministerial type. Maybe there should be a police to say, what do you believe in?

I believe in the white government. I believe that the white government should be the prophet of the people.

[Speaker 3]
And can there be a referral system?

[Speaker 1]
Yes, yes, yes.

[Speaker 3]
Can there be a referral system?

[Speaker 1]
Yes.

[Speaker 3]
Knowing how this one works? Yes. The areas of specialization, and this one.

So that will be fair. There is no change. What I am seeing, I think I need to refer to this traditional.

[Speaker 4]
And it will work, because now we have both sides have the patient's notes how they are tackling from both sides. We all have the information. So we are doing this part, and you are working with us.

If it's just about the antimicrobial resistance, we are trying to tackle it. So this is how we are managing. We want to avoid this.

And I am seeing how you are managing. So we are working together. There won't be any clashes.

[Speaker 3]
And can there be a possibility where we say this is another hospital, where this was, and that room is for the traditional healer?

[Speaker 4]
I think it will work. It can work.

[Speaker 1]
Yes, it can.

[Speaker 4]
Well, I think there is a I don't know how to phrase this. But there is a lagging when it comes to the medical professions. Because we think everything has to be by the book.

But we say it's medical practice. Practice meaning no one knows fully how to manage. Because there are things where we say etiology of the disease, we just write idiopathic.

We don't understand how it came about. What's causing. Meaning that it's a practice.

Yes, in this traditional healers, we should have grace for them and be like they are also doing their practice. So now let's work together and incorporate each other. Yes, I have the scientific knowledge.

I do the lab work. But they are doing their own work also, which is relevant.

[Speaker 3]
Like the treatment of wounds. They can apply the medicine. We used to apply these traditional medicines when we were young for treating the wounds.

But once they see that they are failing to achieve the desirable results, they can say I think it's better to refer.

[Speaker 1]
Some even say this is not spiritual. On their assessment they say this is not spiritual. Please go to the clinic.

[Speaker 3]
Go to the clinic. That referral system should be established.

[Speaker 1]
Yes. People should have a relationship with no one looking down on anyone. No.

Because it's not true. Yes. It's not true.

It's true. Yes. This could be a spiritual intervention.

For example, to take your mother to the hospital, and if the doctor is sick, then you can tell her to come to the hospital. But when you give the medical register to another patient, they will not be able to see the baby. I am not sure what to say.

[Speaker 13]
I am sorry.

[Speaker 4]
A lot of people are going to Chile.

[Speaker 1]
Yes, a lot of people are going to Chile.

[Speaker 4]
What do you think will happen? What will happen is...

[Speaker 1]
Orientation. No, I think orientation is not going to help the youth of Chile. I am a senior, I am not an oriented person.

Chile is not going to have a patient. It is not going to have a patient because they don't know what they are doing. But what happened is, I could not open the door.

The patient could not open the door. They gave me the receipts of the services that you have received. The patient and I received.

[Speaker 4]
That is the warning that my sister gave you?

[Speaker 1]
No, no, that is not the warning. Now, we are going to do. [redacted name], because it was midnight, and there was no BOP, material, [redacted name] gave me a phone call and told me to call my sister.

She told me to deliver.

[Speaker 12]
BOP, BOP.

[Speaker 1]
Yes, BOP. Chiremba told me that there was no receipt. That was not the issue.

But he went on telling me that there was a BOP. I did not know what to do. I did not know what to do.

So, I went to Chiremba. The office of the secretary told me that there was no receipt. That is why.

He did not know. That is the reason. I did not know.

I went to the hospital. The hospital gave me an X-ray. They gave me a BOP of two receipts.

They gave me a BOP of two receipts. They gave me an X-ray. They told me that I was going to die.

They told me that I was going to die. because he died 5 years ago, but his machine was down. So he had to do an X-ray and he died.

I don't know if he was forced to do it, but I don't know.

[Speaker 8]
I don't know.

[Speaker 1]
I don't know what happened to him.

[Speaker 14]
I don't know.

[Speaker 1]
I don't know.

[Speaker 4]
Our aunt is... Our aunt is still here.

[Speaker 14]
As a matter of fact, I understand my daughter. She was going to school.

[Speaker 1]
She was going to school, but she had no money. She could no longer afford school or college, because she had no money. The blood tests are still going on.

The blood tests are still on. But the blood investigations have gone from the machine to the blood sample.

[Speaker 4]
But the blood investigations have gone from the machine to the blood sample.

[Speaker 1]
If the blood doesn't come out, it will be difficult for the patient to be given a shot. It's not that simple. My daughter needed to go outside.

She can't stay in her bed. She can't take the medicine.

[Speaker 3]
Well, coming back to how they better work together, they should understand, not looking down upon each other, there should be mutual respect, my area has no competency, so they should work together, discuss, that and so forth, it would be good, and achieve our benefit from the expertise, it's only good sometimes we don't want to listen to the teachings from the traditional healer, and so forth, if there is that mutual respect, understanding, then there will be an opportunity of teaching, exchanging ideas, exchanging knowledge, so

[Speaker 1]
that, and if these two work together, there is no cheating, yes, there will be no cheating, everything is transparent, because we find that some they claim to be doctors, but they go behind consulting the traditional healers, some also say we don't go to the clinics, but yet behind their followers back, they go to the clinic and seek medical attention,

[Speaker 3]
yes, you nurses, ok, health workers, there was a time, I think it was [redacted name], they are working in hospitals, taking people from the oncology department and referring them to the [redacted name], and so forth, and so forth, and so forth, affecting the attention of the patients, but because we wanted money, it was there, there was monetary aspect.

[Speaker 1]
Out of discouragement, you give them, pay bills.

[Speaker 9]
Here you go, here you go, here you go.

[Speaker 8]
They want money, you people.

[Speaker 2]
The relationship between conventional and traditional medical practitioners, what are the contributions and challenges of trying to integrate traditional medical practice, especially around the IHOP, but I just want to give you another input or stimulation around this. This is one thing that appears in their research, that is traditional medical practitioners as a connection between communities and healthcare. So two areas here that I found quite interesting is, the first area is the question of one health status and identifying disease outbreaks.

Traditional medical practitioners can be, according to the literature, particularly effective in monitoring disease outbreaks. Again, because being closer to the potential patients, being of course more numerous generally, also being trusted in the communities. So they live within the communities and are likely to be one of the first to know if there are any new disease surfaces, for example, comorbidities, TBs.

We also have the potential that is that open lines of communication between traditional medical practitioners and the community of conventional medical medicine, including of course pharmacists, nurses, doctors, medical doctors, could tremendously improve surveillance. And health officials must include that traditional medical practitioners in educational outreach to doctors, but also to other health practitioners, and be trained to know what information you should request from traditional medical practitioners. So this kind of, how do we work together?

The traditional medical practitioners could maybe be taught why, what, when, and how to report unusual symptoms in their patients to the health system. But of course this demands quite a lot of trust and a good relationship. So the suggestion of using, for example in the literature, the suggestion of using checklists and having these pictorial guides to symptoms, diseases, and modes of transmission, and facilitating communication between traditional medical practitioners and the community has potential.

I was talking to colleagues who are doing research in Zambia around antimicrobial systems, and they were using this more like checklists, and especially pictorial guides, like pictures. Like for example, do you have, they have pictures of leaking sewage systems, of how, like things that could indicate risks of water and sanitation quality that could be driving infection. You could also, they were also looking at, for example, solid waste.

They were looking at the presence of packages of antibiotics being discarded in the environment, in communities, to know that there is a high usage of antibiotics. And looking for, for example, expiring dates to see, okay, it's not just that there is a lot of antibiotics being used, but they are also expired, or they are an unusual mixture of it. Because it can sometimes be hard to know, to name the antibiotic.

But if you have pictures of it, it can be easier to identify what is going on. And to relate to that, I have colleagues who are working with that, who are working with research communities in Latin America around antibiotic use. And they were also using this kind of, they were going to local pharmacies and buying different antibiotics.

And then when they met communities, they said, okay, which of these do you recognize from your community? Do you recognize this? And they could look at the packaging and the actual, like, list of packs of it and everything.

So it's one way of not just being stuck in the kind of naming of things, but also using pictures. So that's one side of it. And then, of course, as we've talked about throughout these workshops, it's very important to, for countries to aim up, is the question of avoiding disease outbreaks.

Like, if people don't get the infection, they generally don't need antibiotic after that. So that's one side that I find interesting in the contribution. The other one is, of course, another central topic that we've talked quite a lot about, and that is, of course, compliance and the behaviors of interest.

And patients, according to the literature, have been argued that they listen well to the contribution of medical practitioners. There's trust. And that gives them also the potential to make a big impact when patients complies and manage that with good collaboration for, like, conventional medicine or antibiotics.

So I think this is also an important point relating to, we were talking in the morning about the, on the African countries, which of course is diverse, and generally, according to the WHO, the general relationship between, you have about 200 to 400 people to one traditional medical practitioner, while in general, you have 20,000 people to one conventional medical practitioner. So that highlights that through medical, through conventional medical practitioners, we have an opportunity to also reach patients who miss or don't have access to conventional medicine and the health system. So maybe that also includes, for example, assisting patients who need counter-infection before it goes to the stage of needing antimicrobials, or that they can get a shorter antibiotic course than they would otherwise need.

Again, this kind of dynamic where we have in AWOL between access to antibiotics and access of antibiotics. And an example I found interesting that is, of course, talked about a lot, is the example of TB, the growth that can occur on a positive patient's head, the medication, everyday they don't need their courses. But the challenge that we talked about before also is around that it might be six to eight months of medication, and you need to then both have the compliance of taking that medication, but also having the access to that medication.

So I was looking for studies here in Zim on this, of the traditional medical practitioners in relation to this. Didn't, unfortunately, find so much. Maybe you have more insight on this.

But I found a study a few years ago that said that 89 in South Africa, that 89 of those supervised, for some of those supervised by traditional medical practitioners as part of the project, completed their treatment course for TB, while supervised by other volunteers, of course not conventional medical practitioners, but volunteers in the community, was 67%. So it's a significant change. And interestingly enough, they also, during that same study, saw that the death rate of traditional medical practitioners, patients, was two-thirds lower.

So it was significantly lower. So it was kind of interesting that these are two kind of, in my mind, basic literature opportunities for how could traditional medical practitioners contribute to their kind of addressing both the emergence of the need for life-guarded use, but also for compliance and following through on antibiotic courses, and avoiding, of course, further infection. So with that in mind, I want you to think a little bit and discuss how these questions benefit.

If this is the, it could be one of the, a couple of the opportunities, what are the necessary forms of providers? We talked about that part of the opportunities, but how are we going to do that? What are the kinds of collaboration between conventional, traditional medical practitioners in their work?

How are we, how are that relationship going to look like? How are we going to relate? Because, as you highlighted before, there is tension.

How can we negotiate for the good of the public health? And how can communities in turn then support that? Because it's not just the traditional and conventional medical practitioners.

Communities can also be part of it. So how can they support an integrated health system of conventional and traditional medicine for residents? What are the roles?

We talked about that before. Who is responsible for it? It might not just be the medical practitioners themselves.

And finally, how could an integrated health system support community health as a whole in a nursing home? What would it be like to benefit? What are we striving for?

So those are the three questions I want you to think about and discuss. For the next 15 to 20 minutes.

[Speaker 1]
Thank you.

[Speaker 13]
Thank you.

[Speaker 9]
Thank you.

[Speaker 1]
Thank you. Thank you.

[Speaker 9]
Thank you. Thank you.

[Speaker 1]
Thank you.

[Speaker 8]

Thank you.

[Speaker 4]
So in terms of collaboration, what terms can be put in place? I think the terms they need to consider so that we can respect both parties and no one will feel undermined. So the terms they have to consider, the core beliefs of each system, whether it's the traditional practitioner side or the conventional medicine.

There shouldn't be a breach of the terms. Something that will respect both sides.

[Speaker 3]
Yeah, and still that referral system, if anyone understands that traditional medicine is there to stay as well as the conventional. But there should be a link between the two. No one should look down upon the other and so forth.

So they should collaborate for the benefit of the patient. Because our patients are coming from the community where there are traditional healers. They are ferrying to hospitals where there are the conventional.

But this, for the benefit of this patient, understanding his or her environment, the two, if the patient sees the traditional healer there, the conventional, then he can feel that I'm getting holistic health. So there should be that. And because the government registered the Zimbabwe National Traditional Healers Association as another supporting group in the health care system, then there should be a provision where these two should work together.

[Speaker 4]
Because I'm sure they also go under some sort of exams or some sort of assessment for them to register their practice. So if that's done, then it's been competent enough. So we really need to acknowledge that.

[Speaker 1]
But how do you measure competence in terms of traditional medicine?

[Speaker 4]
The results.

[Speaker 1]
Yeah, the results. You're basing on the results. Meaning that maybe there's a need to take a sample.

So we take a sample of a certain test on a certain day. We take a certain team of people. We sign our consent.

We are given a stipulated time. Our traditional practitioners, we are given a stipulated time. But such a wound should heal in this period.

When you got all the results. Then I think, if I put it in a respect from all the... Yeah.

From all the parties.

[Speaker 4]
I feel like the other challenge that we have when it comes to traditional medical practitioners is that Zimbabwe is mainly a Christianity... I have thought about that. It's a Christianity country.

Yes.

[Speaker 3]
75%.

[Speaker 4]
75% of the people.

[Speaker 1]
But yet 75% of those people that claims to be Christians, they are not.

[Speaker 4]
That's the challenge that we have. That if someone is going to the traditional medical practitioner...

[Speaker 10]
You know the graph? Sorry for that.

[Speaker 4]
We should make sure that traditional medical healers shouldn't be a religion. People should access it, whether you are Christian or not. Everyone should be open to go there.

[Speaker 1]
Regardless of your religion. That's where I was driving to.

[Speaker 4]
We need to push that agenda. That traditional healing doesn't mean that you are believing in something that is not Christian. It shouldn't be linked to your religion.

[Speaker 1]
They associate tradition with witchcraft. But I noticed... I noticed that when I was a sister in church in Zimbabwe, there was this patient who was bedridden for some time.

This patient was her first cubicle. Her first bed, bed number one. So I met him in Zimbabwe.

Do you know where that is? Again. Do you know where that is?

She was coming from very far. So it took them time to get there. They asked for permission to take their patient.

They didn't have permission. So I told them that we can't give them medical advice. Because it's inhumane to disturb them.

Unless we are saying, that's not our own best care. But this patient, we can still do something. So what they did now is, they asked my children, do you permit us?

Because that's your belief. We respect that belief. What they did there, in the following day, I saw him lying on the floor.

He was breathing. He was rising. He didn't have any migraines.

But he was lying there. I asked him, after 30 days, he said this was more spiritual.

[Speaker 3]
It's a spiritual sight.

[Speaker 1]
This was more spiritual. So, like you were saying, I don't know what we can do to measure. To measure the services that we render.

So that each and every party is satisfied to say, even if I go to the traditional healer, I will still get the same results with the conventional. So that all the parties get the same respect.

[Speaker 4]
When it comes to the community support, I think that's one of our main challenges that I was talking about, the religion aspect. But once everyone understands that traditional healers are there to heal you, both the spiritual, it doesn't matter what you believe in, whether you're Christian, whether you're Muslim, traditional healing is important. I think we can get the support from people.

I don't know how we can address the religion issue. But once people are no longer, what do you call it, when your mind is closed. Closed-minded.

[Speaker 1]
So which terms would you use? What are the necessary terms? For collaboration.

For collaboration between them.

[Speaker 3]
You could say it all. Yeah, because you'd find in some homes, in some families, there are some people who get sick. Just because within the family there is either an avenging spirit, which is trying to manifest or to convince, to show the people that there is something they did and it's an avenging spirit, and so forth.

Because when you admit this patient, the temperatures are normal, you take blood, everything you test.

[Speaker 4]
Clinically, the patient, according to the books, it's normal. But when you look at the patient...

[Speaker 3]
When you look at the patient...

[Speaker 4]

The patient is sick.

[Speaker 3]
The patient is sick, and so forth. This is when, you know, and mind you, if our people have a sick patient, before they come to see the patient...

[Speaker 1]
They'll start consulting. You say that the spiritual healer or the traditional healer...

[Speaker 3]
They start by consulting. It's just a sickness, only without something behind. Then they hear that, no, there is this one.

And going to, coming here, you are doing investigation after investigation, everything coming normal. Then this is when they say, ah, we kindly request for a discharge of our patient. Then they take that patient to a traditional healer.

The following week, he comes back. How much is my bill? Walking by himself, and so forth.

And it's happening. It's really happening.

[Speaker 1]
There was a doctor, C4, C3. We would usually complain when we were on night, to say, there is this movement that we hear in the second floor. Then, those that are in the second floor, they would say, there is this movement that we hear on the first floor.

So, when the doctor came, he was like, he was very irritated. He would say, He didn't go for a tour. He didn't go to any stage where he could talk.

He said, I don't want to go to any stage where I can't talk. I don't want to go to any stage where I can't talk. I don't want to go to any stage where I can't talk.

[Speaker 13]
I don't want to go to any stage where I can't talk.

[Speaker 1]
I don't want to go to any stage where I can't talk. I don't want to go to any stage where I can't talk. I don't want to go to any stage where I can't talk.

They thought nomadic people can't talk. I didn't go. I didn't go.

I said to myself, I will go out in the fields and share the news with other boys. The other boys were in the supermarket.

They were not looking for me anyway. The next day I saw the news that the boy was arrested. I thought, how can he be arrested?

I thought he was just a kid. I didn't know what to say. I was thinking, what if he was arrested?

No one commented that night. I didn't know what to say. I was around the house, and we were talking.

We were talking and talking and talking. But he is late. He is late to his work.

And he has no money. Why me? I always ask, I always ask what is in the Bible?

There is a history of Jesus. I used to go to see him. But I went to see Abraham.

It's controversial.

[Speaker 3]
It's controversial. That's why I was saying, going to a traditional healer, majority of people go there during the middle of the night.

[Speaker 1]
They don't fear of the community.

[Speaker 13]
They don't fear of the community. They go there at night.

[Speaker 8]
They go there at night.

[Speaker 1]
They go there at night. They go there at night. They go there at night.

They go there at night. This is happening. We only wish we could die one day and we could run up a chain.

Exactly.

[Speaker 3]
Fear of the community. That's basically what they do. During my training as a hostman, I transfused the three members of Jehovah's Witness.

[Speaker 1]
Three? They don't want to be seen on transfusion. But according to their religion, they don't believe in transfusion.

But they can do it behind the other colleagues' back. So in a way, transparency.

[Speaker 3]
Transparency. The religion. These members, three people, three patients who belonged to Jehovah's Witness, they don't want any blood transfusion.

But those people were dying and I said, tomorrow you will be dead. I know I fear the people who come to see me, but no, it's not a problem. We can transfuse you from 10 p.m. when no one is around. And by 3 o'clock we are done. Then we remove everything and put up a drip. And that was that.

So here in Zimbabwe, people still look down upon the traditional healers. They normally visit them during the middle of the night. They don't visit them during the day.

And yet they claim Zimbabwe has 75 Christians. And yet there is 50% of the Christians who believe in tradition. So they are in between.

25% are staunch Christians, they don't mix. Again, 25% are staunch traditionalists, they don't mix. But the 50% they practice both and so forth.

So if there is that understanding where they can work together, do it in the open, then we are home and dry. So that integration will be very, very important.

[Speaker 1]
Maybe if they could say traditional is not a religion.

[Speaker 4]
Traditional medicine?

[Speaker 1]
No, it's not a religion. You are free, like the way we come to seek medical attention. So everyone is free to choose where you want to go.

[Speaker 3]
And on our part, during our training, when we are on the road of attachment, we would visit a traditional healer, a faith healer. Those people would visit them, know what they are doing, so that you would respect their areas of profession. We visited them, and when you see a patient putting on something on his or her hand, the threads and so forth, you should understand, don't say, I don't want to say that, no.

You should understand where they are coming from.

[Speaker 1]
Like there was this mental patient, the mental ill patient, who came, the investigations, like the... Yes, it was done. Nothing came of it.

It was normal. Then this patient was given the highest dose, the CPZ highest dose, which is 1,200.

[Speaker 9]
The moment we gave that patient... 200 for women.

[Speaker 1]
200 for men. The mental nurse, they say you can go out with that.

[Speaker 4]
No, not assisted dose.

[Speaker 1]

What we did, he was given, and we had to put him in the seclusion. The next minute, we were like checking on him to see, is he still okay? He was even making more noise than before.

Then we were wondering, then the father came, the father came. He said, with all due respect, can I please take my son and go home? There are no drugs, he has never abused drugs, but I know the problem.

Then he was like, when we were looking closely to the relationship, he wasn't his son, but it was the sister's son. But he didn't know the clan, he wasn't aware of his clan. So he was like saying, it's because we don't know his clan, but now the sister is late.

But we have done our consultations. And we were told, as soon as he gets to his clan, he'll be okay. And I will bring him, next week on this date, I will bring him if anything.

Even if he gets better, or if he gets worse, I'll bring him back. They went away. Ask [redacted name] he'll tell you. They went away. And the following day, this guy even came back to say, how are you?

[Speaker 9]
And we were like, The moment, they never gave him anything.

[Speaker 1]
We just went and met these people and it was done.

[Speaker 2]
We are talking about the relationship between traditional intervention and medicine. And of course, as we have seen, there is an emerging or ever-present question.

[Speaker 3]
The question I want to just pose to you is also, has this tradition borne out of our mother tongue, which is not pure Christian, not pure traditionalist, but it's like the notion of health.

[Speaker 2]
The notion of health, not only in the traditional medical practice and in the conventional medical practice, but is it different ways of kind of knowing this? That's the question I'm posing to you. For example, this conventional medicine, as a doctor, you were just listening to that discussion here, and in both groups, you can get a patient, which, biomedically, all the tests are fine, but there is some, you look at it, you have that experiential measure, you look at that, you talk to them, and they are not fine.

So there is different ways of knowing health. The traditional medicine is also being that it is a measure of being able to live the life, you have to reason the value. It's another way of measuring health.

So I just want you to reflect a little bit together, before lunch, we have one more thing after that, is to think about how do we draw these kind of knowledges, how do we relate, how do we use it, especially within AMR education, these kind of different ways of knowing health. Because, of course, it is this experience of health and unhealth, and the tension that we have in the previous workshop has of course been that, quite often, patients are feeling well

enough to start medication. And that can, of course, drive disease and AMR. So how do we work, we talked about the absence of integration, but how do we work and draw on these kind of ways of knowing health?

So let's talk a little bit together about that, and I will check out in the lunch.

[Speaker 4]
I think the tension is because we are undermining the other aspect.

[Speaker 9]
It's the tension, but also looking at different things.

[Speaker 3]
Yeah, we undermine. Yeah, we are undermining. After 1,200 CTs, 200 are sent up and go beyond 200.

Yeah, so that one we have been discussing. We have to respect each one's area of specialization. Don't look down upon the other.

And you would find that in modern medicine, it seems there is a tug of war between the modern medicine, the conventional, and the traditional. And something they try to look down upon the other, especially the conventional, they look down upon the traditional. Even the Christians, they look down upon the African traditional religion.

They try to look down upon that. And yet the fact that it's there, we can modify it because you still benefit from that traditional medicine. I know the traditional medicine, people do believe, yes, we have got a men's clinic for the dysfunction, but the traditional healers, as they are doing better, as well as the STIs, those areas, those two areas, they do treat very well.

[Speaker 1]
Even the women in terms of fertility. Fertility, fertility problems, they do, you know. Even the mental cases, they do.

[Speaker 3]
Yes. So we need, who can bridge the gap? Who can make the two groups understand each other and respect each other?

That's where the problem is. So that we don't visit traditional healers, practice traditional medicine during the middle of the night.

[Speaker 1]
I think you should not say, she looked down on my traditional healers. Even in the Bible, they are there.

[Speaker 9]
Yes.

[Speaker 10]
Moses, Moses.

[Speaker 13]
Moses.

[Speaker 3]
Moses proved to be superior than Pharaohs.

[Speaker 4]
About the discovery of the penicillin, right? I was just trying to make sure my history is right. So, his name was what?

Fleming. He discovered the penicillin, he was growing something, there was a mold, right? Then stuff, this was stuff, right?

Then he noticed that with this molding that was growing, there was another microorganism preventing this mold from spreading, right? Which is the penicillin, another microorganism. So he now purified, obviously now it's now a purified microorganism, it doesn't cause the disease, but it was like these two things, this was just a discovery.

So even the traditional medicine, they found a way, like how can we use whatever the herbs that they have to counter, I don't know how to explain this, but it's like the medicine and the health, it's with us. So we are doing the scientific research and what, but these people, the traditional people, we haven't gone into the traditional medicines to see, so how are they treating these wounds with their traditional medicines? But the traditional healers, they know, they've seen it.

For example, I'm saying, this was, it was found by mistake, then he noticed, oh, we can actually make a drug out of this, it can prevent these antibacterials from growing. So even from the traditional medicine, they may not understand scientifically what's going on, but they know if I use powder A on this type of wound, it's going to heal. So it's a lot, I don't know, we really need to just respect each other.

[Speaker 3]
That system, for your own information, there is a Department of Organic Chemistry that use it. I remember during the time when it was run by Dr. [redacted name], they would say bring as many traditional medicines you know, then they would separate, they would separate and come to know what it contains, and that time they claimed to treat TB, and some of the cancers that use it, yes, they are now separating the traditional.

[Speaker 1]
Like what they are doing now on Moringa, you know Moringa? I'm told the scientists have picked that the... The Moringa tree?

Yes, it has antibiotic properties, anti-inflammatory properties, so now maybe they are now trying to extract the... Study. Yes, study to then further come up maybe with capsules or something.

[Speaker 4]
The first one was the traditional medicine. Yes, the traditional medicine, yes.

[Speaker 1]
Reaching the depth. So who then does bring it together?

[Speaker 10]
I saw, left it to the government.

[Speaker 1]
Yes, the people who regulate the both, they need to bring both parties together. But you know those policemakers are the ones that are more into traditional than conventional, but for them to legalize it and say everyone is free, like the free access to clinics, they don't want it. Why?

I don't know. Maybe they feel they are going to be weakened. We are as young as...

I shall not be wasting time. Yes. Yes.

No, I think it was not able to get it.

[Speaker 3]
It was not able to get it. Competition. It was not able to get it.

And mind you, if you get to a traditional healer, they try by all means, you know, even a root of a tree you know, and they try by all means removing this and that and that so that you can't even recognize what the root is. Because they don't want their medicine to be known by others.

[Speaker 1]
And some people end up dying. They don't even have a root in their mouth. It is the root.

Yes. It is the root. Like I know...

They will get it. They will revive it. They will renew it.

They will relocate.

[Speaker 13]
Yes, yes.

[Speaker 1]
They will get it.

[Speaker 13]
It will spread again. Yes.

[Speaker 1]
Wow. Yes. But it does not go away.

[Speaker 9]
They will stay in the area. Yes.

[Speaker 1]

They will stay in the area. But some people will stay in some places. Yes.

I'm not good at it. It's not bad. I've had to learn a lot since childhood.

You know, the Chinese music. Before you learn it, you may think, no, this is good, and this is very good. But once you learn it, you know it's not good.

[Speaker 10]
Yeah, I haven't tried it yet.

[Speaker 4]
So I'm trying it. Do you want to try it? I don't know if that's what it's called.

But then I'm a mom. So I just said, these days, they don't know. I mean, I'm just...

I mean, I should have my papers. I should have data. But I don't know how to do that.

I'm not a teacher. Then, but... I went to Farmtown with my mom.

So, to my mother, I'm going to farm till I turn 10. I'm going to go back to my grandma one day. I just want to get some money.

[Speaker 3]
I am a bit afraid, I don't know where to go.

[Speaker 1]
I don't know where to go. Can you help me? Can you help me?

I don't know anything. I don't know anything.

[Speaker 4]
I don't know anything. That's it. I am not going to buy a house here.

I am going to go and live with my family.

[Speaker 11]
I am going to live with my family. I thought I would be able to find a good job.

[Speaker 2]
Good.

[Speaker 11]
I have no money. I have no money. I have no money.

I have no money.

[Speaker 2]
The discussion or the question I want you to write a little bit about is, how in all the traditional medical conditions that we knew there, integrated with the conventional medicine approaches. Coming back to this, do you mind that? How would it, how would it affect the purpose of the emergency?

Who will be responsible? Doesn't she? And who are those?

And the emergency is how, and where the emergency is happening. Not only this discussion that we've come up with, I think all of the media that is How are we going to practice MR education at an integrated health system? Which is the purpose of traditional conventional medicine.

So take a few minutes and write some, do some writing on this. Because it's kind of linking it back to those kind of themes that we work with throughout this workshop series.

[Speaker 1]
Before we go home. Are we doing it individually or we are doing it as a group?

[Speaker 2]
Please do some individual.

[Speaker 10]
Who is responsible?

[Speaker 1]
You over-qualify.

[Speaker 9]
Okay everyone.

[Speaker 2]
Let's just share the group. Let me start.

[Speaker 1]
I'm the most talkative one. It seems. Anyway.

I thought maybe the effects are both negative and positive to the AMR education. Involving the traditional medical practitioners, it could be received in a positive way. Which maybe would help reduce the antimicrobial resistance to the community at large.

But at the same time it can be received in a negative way. Which also might bring people not to be compliant to the drugs. Or maybe to abuse the, because the traditional medicines are easy to access.

And like what you were saying before, to say the traditional practitioner would say, you pay me when you are well. Unlike the conventional practitioners, they would say, for me to treat you I need money first. So it might receive a negative, especially even to the government or the policy makers.

Because they would say the health institutions will run short of clients because everyone will be running there. Because now it's open. It's no longer a crime to be seen getting to a traditional practitioner.

That's how I saw it. How I thought it would affect the MR education. Then who will be responsible for the MR education?

To me, I thought it would start with the policy makers. Then maybe come to the practitioners because they have to embrace this, the practitioners themselves. Before it even gets to the communities.

That's how I saw it. Then who is the target of a MR education? I think everyone.

Because at one time I also need to consult. Even the traditional practitioner also needs to consult. So everyone is a target.

Then where is it happening? It's happening, like I said before, in the communities, in the schools, in the health institutions, in the churches. Wherever there is a gathering, it can happen.

That's what I think. Thank you.

[Speaker 10]
That's the same. Churches, communities, hospitals. We need to target the health practitioners.

The community is responsible. Policy makers, doctors, pharmacists, the traditional practitioners. I'm not sure where it's happening.

[Speaker 2]
It's like it changes when we begin to talk about traditional medical education.

[Speaker 8]
It seems the same. Just that more women and educated women are getting married.

[Speaker 3]
It also, I think it's a plus. Oh God, if I beg you.

[Speaker 9]
My question is, how can a boy have a marriage?

[Speaker 1]
Mm-hmm. Understood. Definitely.

[Speaker 9]
Thank you. God bless you.

[Speaker 8]
Thank you.

[Speaker 2]
Who is going to be responsible for doing that education?

[Speaker 8]

The Ministry of Health. The policy makers, the board of the traditional leaders. Who is in that?

Mm-hmm. It's also happening. Mm-hmm.

Pharmacists as well.

[Speaker 9]
And the politicians. Mm-hmm. The politicians.

Mm-hmm.

[Speaker 10]
He's a jaguar for trade, but a master of none.

[Speaker 1]
Can you speak up? We are straining our ears to hear you.

[Speaker 6]
I was thinking, because of the traditional medical education, because if you remember, there's a slide, where you can see the difference of 87% which is the quality of health, as compared to traditional medicine.

[Speaker 9]
They say it has dots. So the traditional leaders need to be educated so that they can be committed to the place.

[Speaker 6]
When you look at it, there are some people who are more into the traditional type of things, where they are sick, they're ill, they are having problems, they go to the traditional medicine. There are some people who are not strong performers of the conventional art. So the traditional people have an impact.

So they need to be educated because of that, because of the community system there. Yes, I know that I went to a certain part, I think it's been, you know, we were talking about the churches.

[Speaker 13]
Mm-hmm. Mm-hmm.

[Speaker 11]
So we are more into the practice of the traditional medicine, because it's coming from Shabu. The traditional Shabu, part of the essence of the traditional medicine, is being educated about medicine, and it's a community conference. I say yes, it's a community meeting, the ministers there, they're talking about the medicine.

[Speaker 2]
But it's coming from someone who's the ministers there, and the community. It's coming. Any other thoughts?

With the practice of AOSD, how would it look like if we were able to achieve this kind of integrated health system that we are discussing and kind of striving for? How would they want to engage in the practice? What would you take back?

I don't know. Because we're going to put down a lot of effort to integrate this, isn't this? And we also need to motivate it.

[Speaker 13]
Mm-hmm.

[Speaker 2]
To have a sense of, is this worth our while, our effort?

[Speaker 1]
I think after the integration, we could come up with a very healthy nation, because everyone is free now to access help from where they feel like, without any stigma being associated with that.

[Speaker 13]
Mm-hmm.

[Speaker 4]
I was also thinking, how are we going to practice this? Like, once we have now this ideal integration, how are we going to roll it out into the community? We need to involve definitely the policymakers.

They will set out some certain rules or regulations. Then we need to involve the medical board. For example, we have the medical board for doctors, nurses, and the traditional healers.

They will have some strict rules where they are regulating how these things are being carried out in clinical practices, surgeries, wherever the traditional healers are going, to see that whatever the things that we want to be done are being carried out in those areas. Everyone, if they see, whether they see the medical practitioner, they also get to see the traditional healer. We want to see that it's being practiced.

So I think there needs to be certain regulations, like strict regulations, which are monitored occasionally so that people will actually practice these things. We can have an integrated system, but it could be just integrated on paper and not actually happening in the community. So we need a follow-up on that and due to strict regulations being actually put in place so that people can work with that.

That's what I think.

[Speaker 2]
Any follow-up?

[Speaker 5]
Okay, the question is how are we going to practice education in the Master's domain? So, like, we have culturally, basically, in the global cultural domain, we could now, we're now

working with the traditional practitioners. It means we are more likely to succeed on our goals because now we have access to most of the population.

Specifically, if the travel is available, given the socioeconomic status, whereby most of the people go to traditional practitioners, that means we have more access to our population. There will be more compliance given that the recent move to the traditional leaders is going to be easier going through them. Also, give us a chance, because it's not only about antimicrobials, it's also about being able to be ready after.

Given that, most of the time, it requires antimicrobial resistance for the 70% of the practice. So, we have a chance to shed light at the same time to create a new system.

[Speaker 2]
Thank you. Any other reflections on these questions? Okay.

Fine then. So, let's wrap it up then for today, for the workshop, by just if anyone wants to just share the primary takeaway from this session. What did we get out of it?

Did we get anything substantially out of these discussions? What did it look like? Do you feel like you have gained a lot?

[Speaker 3]
Yeah. In the Zimbabwean scenario, we should understand that the traditional medical practitioners are there to stay. And there are some benefits from that.

And we should and the conventional practitioners should not look down upon the traditional. Otherwise, we might miss something. We should know very well that our people, if they have got health problems, they seek help both from the traditional medical practitioners as well as the conventional.

So, if we were to work together, we know the government has managed to register Zinata for the traditional healers, knowing that there is a service which can benefit from. So, it should go a step further by making the two working together. There should not be any tension like you had showed up.

But we should complement each other because it is for the benefit of the patient. And this will serve as a holistic approach as far as the treatment or as far as health is provided to our patients, to our members. No one is left behind.

[Speaker 4]
I think it's been covered. I just think at the end of the day, this discussion has just brought an appreciation of what the traditional practitioners are doing and how we say the medical side, we go to school and we attain a skill. These people also have been practicing and they also go through licensing, meaning they also have a skill that we need to appreciate.

So, if we work together for the benefit of the communities,
