# Supplementary material for: Integrating traditional medicine into antimicrobial resistance education: Community-centric preparedness in Zimbabwe
Source: PLOS Glob Public Health. 2025 Oct 27;5(10):e0005245. doi: 10.1371/journal.pgph.0005245 (PMC12558487; doi:10.1371/journal.pgph.0005245)
Supplement: Attachment — Submitted filename: Responses to Reviewers comments.docx [file pgph.0005245.s003.docx]

**Reviewers comments**

***Integrating Traditional Medicine into Antimicrobial Resistance Education: Community-Centric Preparedness in Zimbabwe***

We greatly appreciate the input from the reviewers and editors in providing valuable feedback on the paper. My co-author and I have now worked through the paper and addressed the reviewer's comments. Reviewers’ comments and our response and revisions are outlined in the table below.

| **Reviewers´ comments** | **Authors´ responses and revisions** |
| --- | --- |
| OVERALL COMMENT: If the manuscript can show how traditional medicine practically and educationally intersects with AMR, it has the potential to make a significant contribution. It contains important ideas. Before it is ready for publication, though, it needs to be significantly restructured, nearly completely rewritten, and subjected to more data analysis, presentation, and deeper engagement with both data and conceptual framing. |  |
| **- Introduction—Requires Major Revision—Rewrite** |  |
| Comment 1: Use an active voice overall. | We have revised the introduction to use a more active voice. |
| Comment 2: The authors make meaningful arguments, and there is a clear focus on equity and integration of traditional medicine in AMR preparedness—which is an interesting perspective. |  |
| I would suggest a rewrite of the introduction, as I have identified several areas for improvement. | The introduction has been rewritten in line with the reviewers´ comments. |
| 1. The introduction jumps between prevention and preparedness without clearly demarcating each point or their interconnectedness. I would restructure the introduction more clearly. | The introduction has been restructured to demark points made regarding prevention and preparedness in AMR education and their complementary character. |
| 2. Logical Consistency and Accuracy—Highlighted below on how microbes develop resistance. | We have strengthened the consistency and accuracy regarding how microbes develop resistance.  “microbial resistance is the result of evolutionary processes, including spontaneous mutations where microbes develop resistance genes independently and horizontal gene transfer in which microbes receive resistance genes from other microbes. While antimicrobial overuse increases selection for resistance genes, these processes are ongoing, which means that resistance will emerge even in cases of strict antimicrobial stewardship.” |
| 3. Repetitiveness: Points regarding prevention’s insufficiency, risks, and vulnerability of communities are repeated multiple times without sufficient elaboration or added nuance. | We have worked through the introduction, reducing removing repetitive points and sharpen the argumentation into fewer more elaborated and nuanced sentences. We have focused on how and why prevention insufficiency in AMR education matters, especially for vulnerable communities. |
| 4. Justification for Vulnerable Communities: The exact reason why these communities would be disproportionately affected by prevention-only approaches is not clear. | We have included sentences that justify the reason vulnerable communities are disproportionately affected. Partly it is due to their higher exposure to infection risk from a lack of access to safe water, poor sanitation. In addition, these communities are less able, due to structural conditions, to access formal health care including diagnostics, medicines and treatment in the case of an outbreak. Through AMR education that includes preparedness, they are better able to identify resistant infections early and get the correct treatments to limit the spread of resistance within the community. |
| 5. The link between traditional medicine and preparedness is promising but not sufficiently justified or explained. | We have expanded on the link, highlighting how, Traditional practitioners are early points of contact for health care need as they often live in the communities and are thus part of local social structures with associated trust on the part of the community. They thus become important as part of the preparedness for identifying, containing and managing resistant infection outbreaks and reducing the spread of resistant microbes and genes. In addition, being embedded in the community allows them to identify persistent, contributing to resistant infection surveillance as part of preparedness efforts. Traditional practitioners are thus well positioned as key actors in developing local AMR preparedness through education. |
| * P3L11-12: The transition to Zimbabwe's systemic vulnerabilities and AMR education "vulnerabilities" is not logical. The previous sentence talks about education, while the latter sets up the system in Zimbabwe; just because there is imbalance doesn't make the analogy stand. I would make Zimbabwe's situation clearer. | We have revised the sentences to clarify the link between the imbalance in AMR education and Zimbabwe’s systemic healthcare vulnerabilities, highlighting the tendency in AMR efforts to focus on healthcare professionals' prescription practices and patients' individual behaviours above structural drivers of AMR. |
| The following sentence, L15, makes the assumption that the inequalities in the overall system are due to educational imbalance. Although this might be the point the authors are trying to make, the way they are making it doesn't hold up to me; I would need more depth. | Revised to: While preventive educational efforts in communities are essential, they may create gaps in AMR education if framed around the notion that resistance and resistant infections can be entirely eliminated. |
| *P3L13: exacerbate | We have revised in accordance with the suggestion. |
| *P3L21–P4L14: I would suggest a rewriting of the paragraph. Where points are made clearer. The authors shift between why prevention is not enough and that it induces risks, then talk about what prevention is and what could happen when prevention does not work. The purpose of this section to me is to convince the reader that prevention is not enough, and that does not translate here. I found myself scrolling up multiple times and trying to decipher the info shared to serve the point trying to come across. | We have rewritten the paragraph to provide a clearer argument that prevention is not enough and that it needs to be coupled with preparedness.  The text now begins by defining prevention and outlining its scope, then explicitly transitions to preparedness with a clear definition and examples. It makes the case that prevention alone is insufficient by explaining the inevitability of microbial resistance, highlighting structural factors such as poverty and rural–urban divides, and showing how narrow prevention framing disproportionately impacts vulnerable communities. |
| *P3L21-P4L3: This seems to be the real reason. I think it should be placed higher within the manuscript; it emphasizes the importance of preparedness in AMR education better and sets the stage better. | Revised in accordance to the suggestion. |
| *P4L6: A standalone sentence does not look good; the reasoning behind AMR education includes not transferring well. Reasoning needs to be structured more clearly. | Revised in accordance to the suggestion. |
| * P4L8-L10: The flow and logic of this sentence are off. It sounds like microbes develop resistance to obtaining the mutations and horizontal gene transfer, whereas in reality these are the ways that they gain resistance; the current wording is unclear and does not convey this message. | Addressed in the responses above |
| *P4L12: Should be surveillance. | We have rewritten the sentence but included the point about surveillance in the following sentence.  As seen in stewardship approaches, surveillance systems, protocols, and prescription management have been the main focus, with less attention given to poverty, housing, water and sanitation, and health system access and quality, especially in the rural-urban divide as critical factors shaping antimicrobial need and use (Dixon et al., 2021a; Dixon et al., 2021b). |
| *P4L12-L14: Would need more detail on how a blind spot is created. | We have revised the sentences as part of addressing the previous comments to strengthen the argument presented. |
| *P4L15-L20: I fail to understand why prevention-focused AMR education limits the capacity to deal with resistant infections, as to my point for the paragraph ending with P4L14. | See the revised section on the linkage…where prevention is still critical cut AMR is an inevitable process of evolution |
| *P4L20: limitations | We have re-written the sentence. |
| *P4L20-21: This point has been made multiple times but lacks the structure to understand. I understand the point that prevention-only education increases risks, but why the vulnerable communities are more affected is not clear to me. | Revised to demonstrate their poor socio-economic infrastructure that creates the vulnerabilities. “These risks disproportionately affect vulnerable communities, which face higher exposure to infection due to inadequate socio-economic infrastructure and are often less able to access formal healthcare, including diagnostics, medicines, and treatment in the event of an outbreak “ |
| *P22-P25: "Lack of sufficient preparedness, medical practitioners, and healthcare services could stand without the tools..." is unclear and awkwardly phrased. Consider rewriting as "Without sufficient preparedness, medical practitioners and healthcare services may lack the tools, protocols, and resources..." | We have rewritten the paragraph and kept the comment in mind |
| *P5L4-L9: The transition is a bit rough and abrupt. A clear bridging sentence would work nicely while narrowing the focus. | We have added a bridging sentence and revised the surrounding sentences to signify the narriowing focus to make a smoother transition. |
| *P5L15: Weird way of citing. I would get rid of it. | We have removed the citation. |
| *P6L3-L5: Needs a logical follow-through on why these questions were formulated. For instance, is it because there are gaps in traditional medicine's role both globally and in Zimbabwe? This should be clearer. | We have added a follow-through on why the questions are forumulated.   Despite acknowledging this potential of traditional medicine, research has seldom engaged with the absence of traditional medical practitioners from national AMR educational frameworks in Zimbabwe, limiting the extent and cultural relevance of national AMR education efforts (MOHCC, 2017). This study addresses the identified gap by exploring how traditional medicine can be integrated in AMR education to support preparedness. |
| I would emphasize the novelty and necessity of the gap your research addresses. This also refers to the lack of a clear justification of research focus. | See the above response which highlight the novelty and necessity of the study addressing the research gap. |
| **- Materials and Methods —Requires Major Revision - Rewrite and add content** |  |
| Comment: The section has foundational content but requires clearer presentation and improved structure. Below are general points the authors should address: |  |
| Clearly outline the exact structure and key activities of the workshops, even if briefly. Simply stating that participatory workshops were conducted is insufficient without describing what was done during them. | We have clarified and described the content of the workshop. |
| Use tables or lists to improve readability, especially when reporting participant characteristics. | We have added a clarifying table of participants |
| Justify methodological choices explicitly, including why participatory workshops were appropriate in this context and how they build on the cited literature. | We have clarified the methodological choice and also made the following addition as justification. “Participatory workshops were selected as the primary method because they enable collective knowledge construction, facilitate the exchange of professional and community-grounded perspectives, and are well suited to exploring complex health challenges such as antimicrobial resistance (AMR), which cut across clinical, social, and cultural domains. “ |
| The section is overly repetitive, and sentence structure is often needlessly complex. Revise for conciseness and clarity. | The section has been revised with regard to reducing repetitiveness, complexity, and improving sentence structure to provide more conciseness and clarity. |
| Conceptual explanations should be placed in the Introduction or Discussion. The Methods section should only describe what was done to generate the data presented in this study. | We have moved conceptual explanations to introduction and discussion. |
| Clearly articulate and simplify the analytical approach. Avoid theoretical elaboration; just state the framework and how it was applied to the data. | We have clarified and simplified the analytical approach. |
| Improve the overall organization of the section to ensure a logical and consistent flow. | We have strengthened the overall organisation of the section. See other responses regarding the section on methodology |
| *P6L9–L11: While the authors refer to previous work, a brief summary of the original method would be helpful in understanding how the current study builds upon or adapts that approach. | We have added details on its previous use “This method has been previously utilised in research in Southern Africa to engage in knowledge co-creation around complex sustainability challenges together with practitioners. Results highlighted how participatory workshops can enable practitioners to contextualise sustainability challenges in relation to their lived experiences in developing adaptive approaches to addressing sustainability challenges” |
| *P6L11–L17: The authors describe the outcomes of the participatory workshops, but not the activities themselves. Please mention what concrete tasks or exercises were conducted with participants (e.g., discussion prompts, group mapping, scenario building). Link outcomes directly to the activities used to generate them. | Revised as follows: Participants then contributed through facilitated group activities, in which they shared practical experiences of AMR challenges in their clinical work, described traditional medicine practices relevant to infection management, and collectively identified opportunities for strengthening community-based AMR education, prevention, and preparedness. Contributions included mapping current barriers to appropriate antimicrobial use, suggesting strategies for collaboration with traditional practitioners, and developing community-level preparedness measures such as outbreak reporting mechanisms, health education campaigns, and pathways for timely referral. |
| *P6L17–19: This content overlaps with the earlier lines (P6L9–L11) and could be consolidated to reduce repetition. | We have consolidated the two sentences to reduce repetition. |
| *P6L24–P7L4: The description of participant characteristics is difficult to follow in paragraph form. I strongly recommend using a table to present participant roles, numbers, gender, and affiliation by hospital for improved readability. | We have added a table to account for workshop participants. |
| *P6L6–L11: It would be helpful to include statistics or descriptors indicating participants’ professional engagement levels with AMR and AMR education, similar to the way their job roles and gender are reported. | We do not have this data readily available |
| *P6L11: There is a shift from passive to active voice (“we”) that breaks consistency. I recommend aligning voice throughout the section—either consistently active or passive—to improve readability. | We have revised in line with the comment. |
| *P6L15–16: It is unclear whether recruitment at both hospitals began simultaneously, or if the dates listed correspond to only one of them. Please clarify this. | See addition “These were held separately at each of the teaching hospitals.” |
| *P6L20–L22: This sentence again states that methods were adapted from a previous study but lacks detail. If the outcome of the method is described, the authors should briefly mention how the adaptation was made or implemented in practice. | We have in the response to the previous comment added details on the previous use of the method and the results of its use in these studies. We also added some detail on adaptation that leads into the subsequent revisions. |
| *P6L23: Please indicate how transcription quality was assured. For instance, was it cross-checked, reviewed by multiple researchers, or validated using field notes? | We have clarified that transcripts were validated using contemporary field notes. |
| *P6L24: “Contemporary field notes” is unclear. Consider rephrasing to simply “field notes taken during the workshops.” | This was revised to: The researchers collected data through audio recordings, their own field notes, and written materials that participants produced during group work and submitted at the end of each workshop. |
| *P7L3: The entire “Analytical Approach” section reads like an extension of the Introduction or Discussion. The Methods section should focus on describing the analytical framework used, not reiterating why prevention vs. preparedness matters. A more concise and technical explanation is needed (e.g., thematic coding, grounded theory, etc.). | We have revised the section to be more concise and technical. |
| *P10L19–20: This sentence is redundant with P10L17–18 and should be removed or merged for clarity. | Most of the sentence have been removed, rest have meed merged. |
| **- Results—Major Revision - Rewrite and add content** |  |
| The Results section raises several concerns related to data presentation, analytical depth, and alignment with the study’s AMR focus. While the topic is important, the current presentation lacks the clarity, rigor, and evidentiary support expected from qualitative research. Below are key areas requiring revision: |  |
| 1. Insufficient Use of Data to Substantiate Claims Across all themes, the authors rely on a limited number of participant quotes (2–3 per theme) from a sample of 25. This raises concerns about the representativeness and saturation of the data. The findings are presented as broadly generalizable insights, yet there is no information on 1) how many participants contributed to each theme, 2) whether these views were shared across both workshops, or 3) the presence of any dissenting or contrasting views. This creates the appearance of anecdotal reporting rather than analytical saturation. | We have strengthened the themes with more quotes from the participants. We have clarified that these themes are representative of the majority views across the two workshops as they are the results of inductive analysis of the participants' discussions. |
| 2. Shallow Thematic Development Thematic categories are introduced but not critically unpacked. Core ideas (e.g., trust, accessibility, community embeddedness) are repeated without deeper analysis or qualification. In addition, it remains unclear how the three themes were derived: were they predefined, emergent, or adapted from previous work? | We have clarified in the Methods and Results sections that the themes were developed inductively from the analysis of participants' discussions. We have also clarified this process in terms of analytical steps.  We have added a broader range of quotes to support the themes. |
| 3. Terminology Ambiguity Key terms such as “community healthcare”, “surveillance”, and “complementarity” are used without definition. For example, “community healthcare” is central to the first theme but remains vague—does it refer to first-line care, traditional prevention, or informal triage? | We have clarified key terms. |
| 4. Overinterpretation and Causal Claims Without Evidence Several claims go beyond what the presented data support—for instance, statements about traditional medicine reducing antimicrobial use or strengthening health system resilience. Quotes provided do not demonstrate behavioral shifts, systemic integration, or measurable AMR outcomes. | We have restricted the results to descriptive findings, added some more detail and depth to these descriptions and moved interpretive parts to the discussion. |
| 5. Underdeveloped Link to AMR Education Although AMR education is the central stated focus, most results remain tangential to this theme. The participant quotes rarely reference AMR education, antimicrobial use, or knowledge-building directly. This weakens the connection between the data and the study’s stated contribution. | We have clarified and contextualised the connections between the expanded results and AMR education more directly. “ While many of the participants’ quotes do not explicitly reference AMR education, it was the focus of the workshops and the quotes reflect relevant social and healthcare contexts for AMR education. Participants discussions were analysed as engaging with prevention and preparedness in addressing AMR, appropriate treatment practices, and the potential of integrating traditional medicine into AMR and disease surveillance, all themes indicating the key role of education in connecting traditional medicine with efforts to address AMR in Zimbabwe. Participants discussions of switching between treatments and the importance of trust and accessibility in for health seeking behavior, highlights how knowledge, relationships and practices impact antimicrobial use and disease treatment. In their discussions, participants situated AMR education as part of these everyday practices and outlined a role for traditional medicine as part of opportunities to strengthen AMR education, responsible antimicrobial use, alternative treatments and building surveillance capacity. The results as a whole can thus be read as illustrating the contextual conditions and points of engagement for developing AMR education in Zimbabwe, more than specific descriptions of AMR education initiatives.” |
| 6. Quote Integration and Transcription Issues Some quotes are grammatically unclear or appear paraphrased. Attribution formatting is inconsistent, and one quote appears to contain a broken or incomplete sentence. | We have ensured that quotes are transcribed accurately and formatted.  We acknowledge that some of the participant quotes in this section contain grammatical inconsistencies, broken sentences, and formatting irregularities. Since the data comes from transcribed oral discussions, the quotations sometimes include hesitations, repetitions, or incomplete phrasing. |
| ***Minor Issues:*** |  |
| Redundancy: Several points—particularly community trust, practitioner embeddedness, and access—are repeated across sections without further analytical elaboration. | We reduced repetition by consolidating similar points. In the Results section we now focus on presenting participant perspectives, while in the Discussion we build on these findings by providing analytical elaboration and linking them more explicitly to the preparedness framework. |
| Speculative phrasing: Avoid phrases like “this shows that…”, “this may reduce demand…” unless these are directly supported by data. | We have revised in line with the comment. |
| Terminology clarity: Terms like “preparedness” and “surveillance” must be anchored in context and clearly distinguished from their use in formal health systems. | We have gone through the text to more clearly anchor terminology in context. |
| *P10L22–23: This is already a given. No need to repeat. | Removed. |
| *P10L24–P11L1: Language is interpretive. Avoid drawing conclusions before data is presented. | This largely has been addressed, but you might want to re-read the changes to make sure they make sense |
| *P11L3–L4: This is interpretive and assumptive. The themes are stated to be identified, but no supporting evidence has been provided yet. | This is about the style of argument. You might want to visit this |
| *P11L6–L10: Phrases like “provides important healthcare” are speculative. If the section is focused on workshop findings, results should reflect participant quotes only—avoid editorializing. Reframe these as descriptive summaries grounded in data. | We have clarified these phrases. |
| *P11L10–L11: Sentence beginning with “As highlighted…” is speculative. A more neutral transition, such as “One participant reflected on…,” would improve clarity and tone. | We have revised in line with the comment. |
| *P11L15: “Referring to ”this”—expand or clarify the quote for better context and readability. | We have revised in line with the comment. |
| *P11L17–18: This is speculative and largely repeats the quote. Rephrase neutrally or remove. | We have revised in line with the comment. |
| *P11L18–P12L1: This functions as a meta-statement and reduces coherence. Rephrase or integrate more smoothly. | Done |
| *P12L1: The phrase “will be early” is overly casual in tone—rephrase for neutrality. | Done |
| *P12L2–4: Highly speculative. The suggestion that two quotes support antimicrobial use reduction is unsubstantiated. This content belongs in the Discussion if adequately supported—otherwise, remove it. | The sentence has been removed. |
| *P12L7: The term “complementary” is underdefined. Clarify whether complementarity refers to behavior, improved adherence, delayed access, or clinical outcomes. At present, the usage lacks analytical depth. | We have clarified that participants framed “complementary” as pragmatic coexistence, with traditional medicine supporting immunity or symptom relief while conventional antibiotics addressed persistent infections |
| *P12L8: The sentence beginning with “This is highlighted…” is unnecessary and redundant. | We have revised in line with the comment. |
| *P12L13–14: Redundant phrasing—streamline. | We have revised in line with the comment. |
| *P13L1–L3: The claim that traditional medicine offers alternatives to antibiotics is not supported by the data. Participants describe simultaneous use, not substitution. Remove or revise. | Rephrased accordingly |
| *P13L3–L6: This is a major extrapolation. Nowhere in the data is it stated that traditional medicine reduces prescriptions, changes behavior, or impacts resistance. Move this to the discussion and qualify it appropriately, or remove it. | The sentence has been moved and integrated into the discussion with appropriate qualification. |
| *P13L7 (entire section): This section does not align with the stated scope of the study (AMR education). If retained, the authors must 1) define what “surveillance” means in this context; 2) provide evidence of actual engagement or referral behavior by traditional practitioners; and 3) clearly connect findings to AMR education outcomes. | 1) We have clarified what surveillance means in this context as response to an earlier comment. 2) we have clarified that the participant discussions and quotations highlight potential rather than actual engagement and referral behaviour.  3) We have connected the findings to AMR education. |
| *P13L8: “Play a crucial role” is too strong a claim given the limited and vague supporting evidence. Revise language for accuracy. | We have revised in line with the comment. |
| *P13L9: The term “AMR and disease surveillance” needs definition. Quotes imply a loose relationship but provide no evidence of how traditional practitioners engage with formal surveillance efforts. | We have clarified the use of AMR and disease surveillance and strengthened the link of the quotes with the potential role of traditional medical practitioners in formal surveillance efforts. |
| *P13L11–L14: The quote is hypothetical (“can be”), not empirical. Authors must acknowledge the speculative nature or provide evidence that traditional practitioners are involved in AMR surveillance. | We have revised in line with the comment. |
| *P13L16–L19: This quote is grammatically broken and unclear. Either correct, exclude, or summarize as paraphrased (and state that explicitly). | Corrected |
| *P13L23–L25: The claim that “traditional medical practitioners play a crucial role…” is a strong overstatement not supported by the data. Rephrase to reflect participant perspectives more accurately or remove. | Replaced crucial with important |
| **Discussion:**  The Discussion attempts to reframe AMR education through a community-centric lens by highlighting the potential role of traditional medicine in prevention, surveillance, and preparedness. While the ambition is commendable, the Discussion suffers from: |  |
| 1. Overinterpretation of limited data: -- The data presented in the Results section of the manuscript are not strong enough to justify claims made in the discussion section. The manuscript uses 25 participants and uses only 2-3 per theme. -- Temper all such claims (such as community surveillance being driven by traditional practitioners or traditional medicine being "key" for AMR surveillance) with conditional or speculative language within the discussion. | We have strengthened the results with supporting quotes and also moderated some claims. Also removed the repetitive sections. |
| 2. Redundancy and verbosity: The discussion would benefit from a clearer structure with content blocks for superior readability and focus, where each paragraph should only make a single well-supported point. | We have limited redundancy and verbosity by creating structure for the discussion. |
| 3. Conceptual overreach: Define key terms clearly. | We have made efforts to define key terms more clearly. |
| 4. Uncritical literature integration: The use of citations at P14L16 and L22 does not support a claim. Mention why the citations are relevant; explicitly use citations only to support the claim and position made. | We have clarified why the citations are relevant and made their use more explicit in our argument. |
| 5. Lack of engagement with limitations and nuance: -- The only limitation is set to be that health practitioners were included. - However, there is no reflection on: -- The low number of people 25 with only two workshops and the strength of the claims being made. -- Potential bias of formally trained personnel on traditional medicine. -- Urban settings skewing perceptions about traditional practices. | We have revised the discussion to engage with limitations and nuance throughout the discussion section. This includes the number of participants, the impact of formally trained health practitioners and conducting the workshops in urban settings. We framed these limitations in relation to future research. |
| 6. Neglect of Contradictions or Tensions: A robust discussion should acknowledge complexity, not just convergence. No mention of possible scepticism towards traditional medicine is mentioned; any divergent views were also not shared. Even if they do not exist, the authors should explicitly state this is a limitation. | We have revised the discussion to acknowledge the contradictory complexity associated with the role of traditional medicine in AMR education. We have clarified the lack of contradictory views as a limitation that we will seek to address in future research. |
| **- Other -** |  |
| *P18L22: Isn't this PLOS One? I suspect the authors have already submitted the manuscript to another journal. Please make sure the manuscript is submitted in the correct format. | We have addressed this point. |
| *Regarding data availability: The authors have provided a 52-page document, which I assume to be the full transcript of the two workshops; however, this is neither explicitly stated nor referenced within the manuscript. The nature and purpose of the supplementary data should be clearly described. More importantly, the data—if intended as a transcript—needs to be processed and presented in a way that supports reader comprehension. In its current form, the raw data is not meaningfully integrated into the analysis. The authors are expected to analyze the data and reflect key insights within the Results section, rather than relying on readers to extract meaning independently. | We have addressed this point by clarifying that “Full transcripts the two workshops are attached to the paper as a supplementary document (Supplementary Material 1). This document represents the raw data used in the analysis and serves as supporting documentation to enhance transparency. The data were systematically analyzed using an inductive thematic content analysis, and key insights have been synthesized and presented in the results section, with illustrative participant quotes included to support transparency and reader comprehension. This is to enable readers to grasp the analysis without requiring direct interpretation of the raw transcript data.” |
